# AET5: A transcriptome-guided molecular generation framework with contrastive self-supervised learning

**DOI:** 10.1371/journal.pcbi.1014703

**Published:** 2026-09-10

**Authors:** Zhikang Yuan, Xin Zhang, Gaoming Lin, Quan Zou, Subhashisa Swain, Yijie Ding, Prayag Tiwari, Shuofeng Yuan, Xiaoyi Guo

**Affiliations:** 1 School of Electronic and Information Engineering, Suzhou University of Science and Technology, Suzhou, Jiangsu, China; 2 Hangzhou Institute of Medicine, Chinese Academy of Sciences, Hangzhou, Zhejiang, China; 3 School of Computer Science and Technology, Zhejiang Normal University, Jinhua, Zhejiang, China; 4 Yangtze Delta Region Institute (Quzhou), University of Electronic Science and Technology of China, Quzhou, Zhejiang, China; 5 University of Oxford, Oxford, Oxfordshire, United Kingdom; 6 Faculty of Computing, Harbin Institute of Technology, Harbin, Heilongjiang, China; 7 School of Information Technology, Halmstad University, Halmstad, Sweden; 8 Li Ka Shing Faculty of Medicine, The University of Hong Kong, Hong Kong SAR, China; Central South University, CHINA

## Abstract

Gene expression profiles capture system-level drug responses and offer a promising basis for *de novo* molecular generation. However, their application is limited by data sparsity and experimental noise, which hinder the reliable mapping between disease-associated transcriptomic perturbations and chemically valid therapeutic molecules. Here, we present AET5, a *de novo* molecular generation framework that conditions molecular design on disease-reversal gene expression profiles. AET5 integrates contrastive self-supervised learning with pre-trained sequence-to-sequence models to learn robust associations between transcriptomic signatures and molecular structures by deriving noise-tolerant transcriptomic representations and aligning them with molecular sequence space. Across the L1000 dataset, AET5 outperforms existing expression-guided generation methods in generation quality and distributional characteristics, while maintaining favorable physicochemical and drug-related properties. We further apply AET5 to generate candidate compounds for SARS-CoV-2 infection and prostate cancer. Molecular docking and dynamics simulations indicate stable target binding, supporting the biological relevance of the generated molecules. These results demonstrate that disease-reversal expression profiles can effectively guide *de novo* molecular generation, providing a general framework for biologically informed drug design under noisy transcriptomic conditions.

## Author summary

Gene expression profiles provide a system-level view of how cells respond to diseases and drug treatments, making them valuable for computational drug discovery. However, directly using transcriptomic data to guide *de novo* molecular generation remains challenging because these data are often sparse, noisy, and difficult to map reliably to valid chemical structures. In this study, we propose AET5, an expression-guided molecular generation framework that designs molecules conditioned on disease-reversal gene expression profiles. AET5 combines contrastive self-supervised learning with a pre-trained sequence-to-sequence molecular generator to learn robust associations between transcriptomic signatures and molecular structures, thereby reducing the influence of experimental noise and improving the alignment between biological perturbation information and chemical space. Experiments on the L1000 dataset show that AET5 achieves improved overall generation performance compared with existing expression-guided methods and produces molecules with favorable structural, distributional, and physicochemical characteristics. Case studies on SARS-CoV-2 infection and prostate cancer further suggest that the generated compounds may have biologically relevant target-binding potential. Overall, AET5 provides a scalable and biologically informed strategy for transcriptome-guided molecular design.

## 1. Introduction

The discovery of drug molecules with desired biological activity and therapeutic efficacy remains a major challenge in biomedical research. Dobson emphasized the vastness of chemical space and its close connection to biological function [1], and Reymond further estimated that synthetically accessible organic molecules may reach approximately 10^60^, only a minute fraction of which has been experimentally explored [2]. Meanwhile, conventional high-throughput screening (HTS) is costly, inefficient, and limited in predictive validity [3,4]. More recently, concerns regarding predictive validity in drug discovery have further highlighted the limitations of conventional screening paradigms [4], and the economic burden of drug development further highlights the need for more efficient strategies [5]. These challenges have driven increasing interest in computational approaches for molecular design.

Artificial intelligence has become an important paradigm for *de novo* molecular generation in drug discovery [6]. Early studies demonstrated that chemically meaningful molecules could be generated through learned latent representations. Representative methods include Variational Autoencoder (VAE)-based molecular design [7], Grammar VAE [8], Junction Tree VAE [9], GAN-based models such as Mol-Zero-GAN [10] and objective-reinforced Transformer GANs [11], as well as sequence- and graph-based approaches including randomized SMILES [12], bidirectional recurrent neural networks [13], and GraphAF [14]. More recently, Transformer-based architectures have further improved the modeling of long-range dependencies in molecular sequences. Building on this paradigm, molecular language models such as MolT5 [15,16], MolGPT [17], and Chemformer [18], have demonstrated the effectiveness of Transformer-based pretraining and generation for molecular design.

Gene expression profiles provide a system-level description of cellular state and naturally connect molecular perturbations with phenotypic responses. Conditioning molecular generation on transcriptomic signatures offers the possibility of designing compounds that reverse disease-associated expression states toward healthier ones. Compared with other omics modalities, transcriptomic data are more widely available and experimentally scalable, and they directly reflect cellular responses to small-molecule perturbations. However, gene expression data are often noisy and difficult to model robustly, especially under limited sample sizes [19], which hinders effective integration with molecular generative models.

In expression-guided molecular generation, early studies explored the feasibility of de novo generating hit-like molecules from gene expression signatures using relatively simple neural network models, such as generative adversarial networks [20], autoencoders [21], and VAEs [22,23]. This line of work was further extended by TransGEM [24], Gx2Mol [25], GxVAEs [26], and SmilesGEN [27], which further demonstrated the feasibility of generating molecules conditioned on biological profiles. Despite this progress, most existing methods still emphasize chemical syntax, molecular properties, or repurposing of known compounds rather than explicitly constraining biological effects during generation. For example, omics-based drug discovery in precision oncology has mainly focused on translational applications [28], while mechanism-driven phenotype screening has often been used for prioritization and repurposing, such as in COVID-19 drug repurposing [29]. Similarly, cross-domain relational methods have mainly addressed drug repositioning [30,31]. At the biological level, prior studies have more often modeled target proteins and biomolecular interactions, such as protein–ligand binding affinity prediction [32] and protein interaction pattern recognition [33], than downstream cellular regulatory outcomes. As a result, incorporating explicit biological-effect constraints, such as gene regulatory responses, into molecular generation remains relatively underexplored.

Another important limitation lies in how transcriptomic features are embedded and incorporated into molecular generative models. Existing expression-guided methods often condition generation through direct concatenation, simple linear projection [20,22–24,34], or VAE-based encoding of gene expression profiles [25–27]. However, these strategies may be insufficient for capturing complex cross-modal relationships between gene expression and molecular structure. Linear layers provide only limited representational capacity and are generally inadequate for denoising noisy transcriptomic signals or modeling nonlinear gene–gene dependencies. Although VAE-based encoders can learn compact latent representations, they are typically optimized to produce smooth and continuous latent spaces for generation. When used as expression encoders, such smoothing and distributional regularization may distort the intrinsic semantic structure of transcriptomic profiles, weaken disease- or perturbation-specific signals, and obscure biologically meaningful regulatory patterns. Consequently, current embedding strategies may limit the biological relevance and controllability of expression-guided molecular generation.

In addition, disease contexts impose different requirements on expression-guided molecular design. Viral infections often involve relatively compact but rapidly changing molecular backgrounds with scarce and noisy data, whereas cancer presents highly heterogeneous cellular states, strong gene–gene correlations, and high-dimensional expression patterns [28]. These disease-specific characteristics further challenge simple conditioning mechanisms and call for more expressive transcriptomic representation learning strategies that can preserve biological semantics, suppress noise, and align gene regulatory responses with molecular generation. Addressing these limitations is essential for improving both the biological relevance and the synthetic feasibility of generated molecules across diverse disease settings.

To address these challenges, we propose Anti-disease Expression T5 (AET5), a framework for expression-conditioned *de novo* molecular generation. AET5 combines a self-supervised contrastive transcriptome conditional encoder with a fine-tuned MolT5 generator [15]. Unlike approaches that directly fuse expression vectors with molecular representations, AET5 decouples biological representation learning from molecular generation. The transcriptome conditional encoder learns low-dimensional, context-preserving latent features that capture disease-specific cellular contexts from expression profiles, while MolT5 uses its pretrained language modeling capability to generate candidate molecules conditioned on these features. Recent studies have also demonstrated the broader value of language models in biomedical research, including data analysis and workflow automation [35].

This modular design improves robustness under small-sample conditions and enables AET5 to adapt to distinct disease scenarios, including viral infections and cancer. In the SARS-CoV-2 and prostate cancer (PC) case studies, AET5 achieved competitive or improved performance over existing expression-guided generation methods across a range of evaluation criteria, including multiple physicochemical properties as well as metrics derived from expert knowledge and pretrained models. These results suggest that decoupling transcriptomic representation learning from molecular generation can improve the robustness of expression-conditioned molecular design across distinct disease contexts.

Our key contributions are:

**Biologically grounded representation learning:** We propose a contrastive self-supervised framework for conditional embedding learning that incorporates cellular context by constructing a high-dimensional representation space of cellular states, thereby transforming noisy transcriptomic signals into discriminative biological representations. The method captures inter-gene dependencies through representation learning and incorporates cellular background as a generative constraint, thereby shifting molecular design from simple data fitting toward biologically grounded conditional generation.

**Simple, robust, and scalable architecture:** We propose a decoupled framework in which the conditional encoder and the generator are trained independently. A parameter-efficient fine-tuning strategy is adopted for the generator, enabling effective learning under small-sample regimes by largely bypassing the need to relearn sequence-to-sequence chemical syntax and instead focusing on modeling the relationship between gene expression conditions and molecular structures.

**General-purpose expression-conditioned generation:** We demonstrate the generality of the proposed framework by applying it to two representative yet mechanistically distinct disease settings—viral infection and cancer—using SARS-CoV-2 and PC as case studies, showing that a single expression-conditioned generative model can produce disease-relevant molecules across diverse biological contexts.

## 2. Methodology

**Fig 1:**
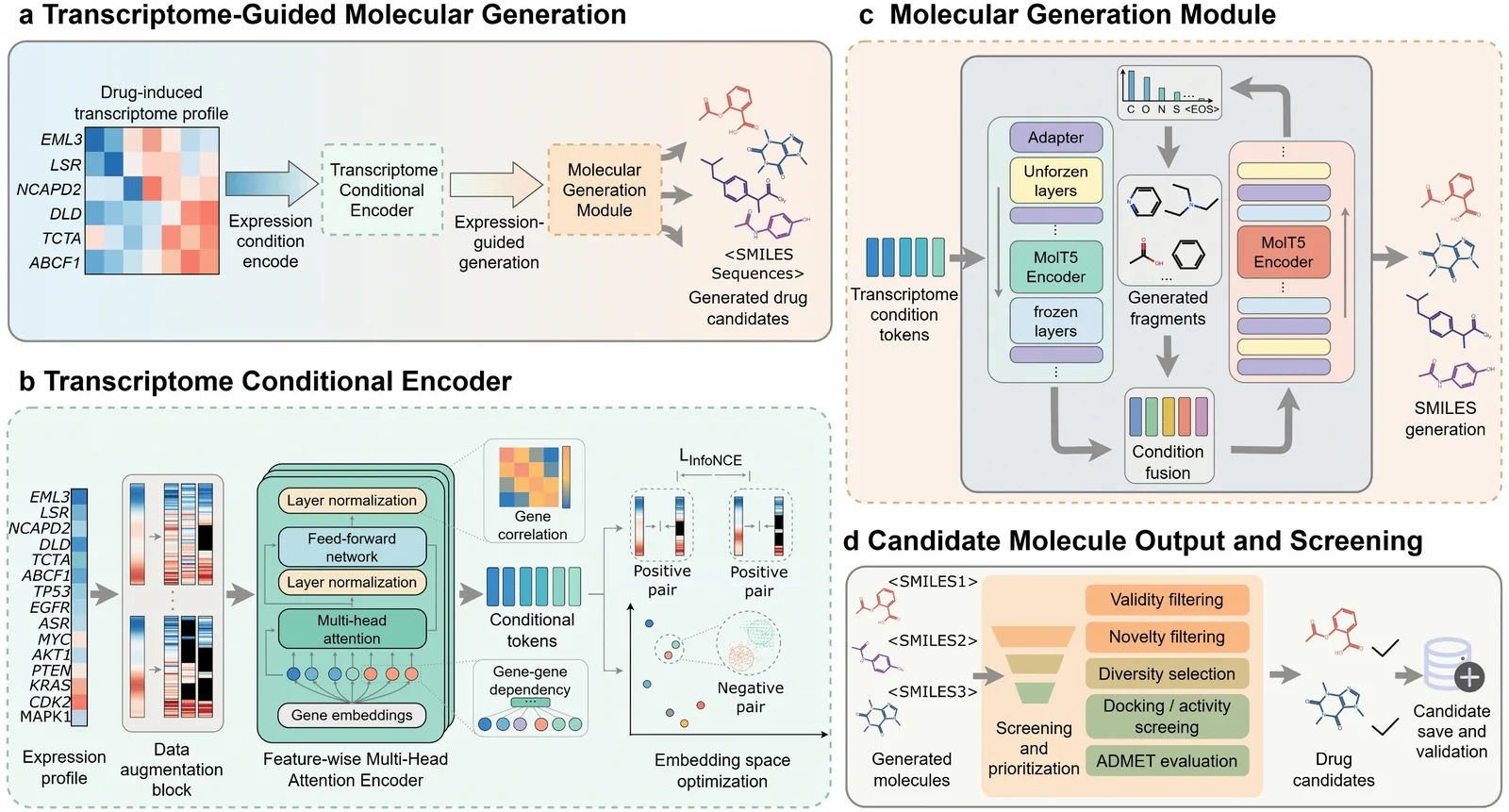
Overall framework of the expression profile-based T5 model for anti-disease molecule generation. **(a)** Overall framework of transcriptome-conditioned encoding and guided molecular generation, comprising the transcriptome conditional encoder and the molecular generation module. **(b)** Expression profile encoder based on data augmentation and self-supervised learning, designed to model inter-gene dependencies and mitigate experimental noise. **(c)** MolT5 fine-tuning strategy for conditional molecular sequence generation, incorporating adapters, selective layer unfreezing, and cross-attention-based condition fusion. **(d)** Downstream screening workflow for generated molecules.

Fig 1 illustrates the overall framework of AET5, which adopts a dual-module design (Fig 1a) consisting of the transcriptome conditional encoder and the molecular generation module, aiming to generate candidate molecules based on expression profiles. The transcriptome conditional encoder (Fig 1b) extracts core features from high-dimensional, multi-locus expression profiles using self-supervised contrastive learning. Experimental transcriptomic data often contain systematic noise, such as batch effects, platform drift, and non-specific probe hybridization. Such noise can distort expression values and reduce the downstream model’s ability to capture true biological signals. To address this, the encoder leverages an inter-feature attention mechanism to capture dependencies among genes and suppress noisy or less informative signals while preserving the global relational structure of expression features. Gene-level expression signals are embedded into a high-dimensional latent space, enabling the model to represent expression profiles on a per-gene basis. Self-supervised contrastive learning enables robust feature extraction without relying on manual labels, thereby providing stable and biologically meaningful conditional signals for downstream MolT5 fine-tuning.

The molecular generation module (Fig 1c), built on the pretrained MolT5 model, uses the transcriptome-derived guiding tokens from the conditional encoder to guide molecular generation. These guiding tokens are first contextualized through the MolT5 self-attention mechanism and are then integrated with the decoder’s current molecular tokens via cross-attention to generate candidate molecular sequences. This design ensures that the generation process is constrained by the biological context embedded in the expression profiles. To efficiently leverage pretrained knowledge while minimizing parameter updates, the fine-tuning strategy uses lightweight adapters with selective layer unfreezing. This design enhances MolT5’s adaptability to expression-driven molecular generation.

Since this task is essentially a conditional text-generation problem, the fine-tuning process applies teacher forcing [36] during training to improve alignment with the target distribution, stabilize learning, and accelerate convergence. To encourage exploration and enhance diversity, the model is trained with variant SMILES, representing different valid SMILES strings of the same molecule. This approach improves the quality and structural variety of the generated molecules. After generation, the candidate molecules are further evaluated through a downstream screening workflow (Fig 1d), which prioritizes molecules with desirable chemical validity, structural properties, and potential biological relevance for subsequent analysis. The source data and code for our study are available at: https://github.com/YuanZhikang-git/AET5.

### 2.1. Transcriptome conditional encoder

Let $x=\left[x_{\text{1}},\ldots ,x_{K}\right]$ denote a normalized drug-induced gene expression profile of *K* genes, where *x*_*k*_ is the standardized expression value of gene *k*. The transcriptome conditional encoder is pretrained via self-supervised contrastive learning to extract robust latent representations that remain consistent under diverse perturbations. To explicitly model contextual dependencies among genes, we treat each gene as a token and apply feature-wise attention across all *K* genes within each sample. Since genes exhibit dependency relationships but no intrinsic sequential order, each one-dimensional gene expression value is first projected into a high-dimensional embedding space through a feed-forward network, and the resulting gene tokens are then processed by a multi-layer attention encoder. In this work, feature-wise attention and the gene-level transformer refer to the same encoder architecture. This design enables the model to capture gene contextual information while mitigating experimental noise.

Two augmented views of ***x*** are generated using a stochastic combination of four augmentation operators: feature dropout (*D*), random scaling (*S*), adaptive gaussian noise (*G*_*adap*_), and structured gaussian noise (*G*_*scruct*_). The augmentation process for the *i*-th view is defined in Equation (1):


$$\begin{array}{c} {\tilde{x}}^{\left(𝕚\right)}=A_{i}\left(x\right)=D_{i}\circ S_{i}\circ G_{adap,i}\circ G_{struct,i}\left(x\right), \end{array}$$ (1)

where each operator is randomly parameterized to create diverse perturbations, and the composition order does not carry specific modeling significance. Specifically, feature dropout randomly masks gene features, random scaling perturbs expression values at the gene level, adaptive Gaussian noise injects feature-wise noise with variances estimated from the training set, and structured Gaussian noise is sampled from a multivariate Gaussian distribution N*(0,****Σ****)*, where ***Σ*** is obtained by scaling and regularizing the empirical covariance matrix of the original data. Efficient sampling is implemented through Cholesky decomposition, and the sampled noise is directly added to the input expression profile.

Each augmented view is then encoded by the gene-level transformer *f*_*θ*_*(·)* to produce contextualized gene representations, which are further compressed by a projection head into sample-level latent vectors, as defined in Equation (2):


$$\begin{array}{c} h_{1}=f_{\theta }\left({\tilde{x}}^{\left(\text{1}\right)}\right),h_{2}=f_{\theta }\left({\tilde{x}}^{\left(\text{2}\right)}\right), \end{array}$$

$$\begin{array}{c} c_{1}=g\left(h_{1}\right),c_{2}=g\left(h_{2}\right), \end{array}$$ (2)

where *g(·)* denotes the projection head and $c_{i}\in R^{dc}$ is the compressed transcriptomic representation used for contrastive learning. The model is optimized at the sample level using the InfoNCE loss [37,38]. Specifically, the two augmented views of the same expression profile form a positive pair, while representations from other samples in the same batch serve as negative pairs. The InfoNCE objective is defined in Equation (3):


$$\begin{array}{c} L_{InfoNCE}=-\frac{\text{1}}{B}\sum_{i=\text{1}}^{B}\text{log}\frac{\text{exp}\left(\frac{sim\left(c_{1}^{i},c_{2}^{i}\right)}{\tau }\right)}{\sum_{j=\text{1}}^{B}\text{exp}\left(\frac{sim\left(c_{1}^{i},c_{2}^{j}\right)}{\tau }\right)}, \end{array}$$ (3)

where *sim(·,·)* denotes cosine similarity, *τ* is the temperature parameter, and *B* is the batch size. By minimizing this loss, the encoder learns robust and context-aware transcriptomic embeddings that preserve inter-gene dependencies while remaining invariant to noise and augmentation.

After pretraining, the transcriptome conditional encoder is frozen and used to map an input drug-induced expression profile ***x*** to a latent conditional vector $c=g\left(f_{\theta }\left(x\right)\right)$. This projected representation is then passed to the downstream molecular generation module as the transcriptome-derived condition for candidate molecule generation.

### 2.2. Molecular generation module

Let $s=\left[s_{1},\ldots ,s_{N}\right]$ denote the target SMILES token sequence of length *N*, where each ***s***_***n***_ ∈*V* is a token from the SMILES vocabulary *V*. Given the transcriptome-derived conditional embedding ***c*** *= g(f*_*θ*_*(****x****))* produced by the pretrained and frozen transcriptome conditional encoder, the molecular generation module generates candidate molecules under the guidance of the biological condition. The module is implemented by fine-tuning a pretrained MolT5 model in a conditional generation manner.

To make the transcriptomic condition compatible with the hidden space of MolT5, the conditional embedding ***c*** is first transformed into a fixed-length sequence of condition tokens through a feed-forward condition-mapping network, as defined in Equation (4):


$$\begin{array}{c} C=Reshape\left({FFN}_{cond}\left(c\right)\right)\in R^{K_{C}\times d_{T}}, \end{array}$$ (4)

where *FFN*_*cond*_*(·)* denotes the condition-mapping feed-forward network, *K*_*C*_ is the number of condition tokens, and *d*_*T*_ is the hidden dimension of MolT5. The resulting condition tokens ***C*** are used as the input to the MolT5 encoder. Through encoder self-attention, these tokens are contextualized into condition representations that guide SMILES generation through decoder cross-attention.

At decoding step *n*, let $Y=\left[y_{1},\ldots ,y_{n-1}\right]\in R^{(n-\text{1})\times d_{T}}$ denote the embedding sequence of the previously generated SMILES tokens ***s***_***1,***_*…,****s***_***n-1***_. The initial encoder and decoder inputs are defined in Equation (5):


$$\begin{array}{c} H_{enc}^{0}=C,H_{dec}^{0}=Y. \end{array}$$ (5)

For each encoder layer *l*, the condition-token representations are updated by the MolT5 encoder block with an inserted lightweight adapter, as shown in Equation (6):


$$\begin{array}{c} {\overset{―}{H}}_{enc}^{l}={FFN}_{enc}^{l}\left(SelfAttn(H_{enc}^{l-1})\right), \end{array}$$

$$\begin{array}{c} H_{enc}^{l}={Adapter}_{enc}^{l}\left({\overset{―}{H}}_{enc}^{l}\right). \end{array}$$ (6)

Let $H_{enc}=H_{enc}^{L}$ denote the final encoder output after *L* encoder layers. For each decoder layer *l*, masked self-attention is first app*l*ied to the embeddings of the previously generated SMILES tokens, followed by cross-attention over the encoded transcriptomic condition representations, as defined in Equation (7):


$$\begin{array}{c} {\overset{―}{H}}_{dec}^{l}={SelfAttn}_{dec}^{l}\left(H_{dec}^{l-1}\right), \end{array}$$

$$\begin{array}{c} E^{l}={CrossAttn}_{dec}^{l}\left({\overset{―}{H}}_{dec}^{l},H_{enc}\right), \end{array}$$

$$\begin{array}{c} H_{dec}^{l}={Adapter}_{dec}^{l}\left({FFN}_{dec}^{l}(E^{l})\right). \end{array}$$ (7)

For clarity, residual connections, layer normalization, and dropout operations are omitted from the equations. Through this encoder-decoder interaction, the decoder attends to both the previously generated SMILES context and the transcriptome-derived condition representations, thereby enabling biologically conditioned molecular generation.

To efficiently leverage the pretrained molecular knowledge in MolT5, lightweight adapter modules are inserted into Transformer blocks, and selective layer unfreezing is applied during fine-tuning. Specifically, most pretrained MolT5 parameters remain frozen, while adapter parameters, the condition-mapping network *FFN*_*cond*_, and selected upper-layer weights are updated. This strategy reduces the number of trainable parameters while improving adaptation to expression-driven molecule generation.

During training, teacher forcing is applied: when predicting token ***s***_***n***_, the ground-truth preceding sequence **s**_**1**_,…,**s**_**n-1**_ is fed into the decoder. The model is optimized using the conditional autoregressive negative log-likelihood, as defined in Equation (8):


$$\begin{array}{c} L_{T}=-\sum_{n=\text{1}}^{N}logp_{\psi }\left(\;s_{n}|s_{1},\ldots ,s_{n-1},C\right), \end{array}$$ (8)

where *ψ* denotes the trainable parameters of the conditional MolT5 generator, including the adapter modules, the condition-mapping network, and the selectively unfrozen MolT5 parameters. During inference, candidate SMILES sequences are generated autoregressively under the transcriptomic condition using stochastic decoding to improve molecular diversity.

### 2.3. Training and inference procedure of AET5

**Alg.** 1 summarizes the training and inference workflow of AET5. The overall framework is trained in two sequential stages. In the first stage, the transcriptome conditional encoder is pretrained using self-supervised contrastive learning on drug-induced expression profiles. For each expression profile ***x*** two augmented views are generated and optimized with the InfoNCE loss defined in Equation (3), enabling the encoder to learn robust transcriptome-derived representations. After pretraining, the transcriptome conditional encoder is frozen and used to extract the latent conditional embedding ***c***.

In the second stage, the molecular generation module is fine-tuned using paired expression profiles and SMILES strings. The frozen conditional encoder provides the transcriptomic condition ***c***, which is converted into condition tokens ***C*** as described in Equation (4). The condition tokens are fed into the MolT5 encoder, and the MolT5 decoder is trained to generate the corresponding SMILES sequence using teacher forcing. During this stage, the parameters of the transcriptome conditional encoder remain fixed, while the condition-mapping network, adapter modules, and selected MolT5 parameters are updated by minimizing the conditional autoregressive loss in Equation (8).

During inference, a new expression profile is first encoded into ***c*** and then transformed into condition tokens ***C***. The fine-tuned MolT5 encoder-decoder generator autoregressively generates candidate SMILES sequences conditioned on ***C***. The decoding process terminates when an end-of-sequence token is produced or the maximum decoding length is reached, and the resulting SMILES strings are collected as candidate molecules.

**Alg. 1** Training and Inference Workflow of AET5

 **Input:** Expression profiles ***X*** *= (x*_*i*_*)*, SMILES strings ***S*** *= (****s***_***i***_*)*;

 **Output:** Generated candidate molecules ***M***;

 **Stage 1: Pretrain transcriptome conditional encoder**

 **for** *epoch = 1* **to** *E*_*enc*_ **do**

  Generate two augmented views for each expression profile;

  Optimize the conditional encoder using the InfoNCE loss in Equation (3);

  Update encoder parameters *θ*;

 **end for**

 Freeze the transcriptome conditional encoder;

 **Stage 2: Fine-tune molecular generation module**

 **for** *epoch = 1* **to** *E*_*gen*_ **do**

  Extract latent conditional embeddings ***c*** from expression profiles;

  Convert ***c*** into condition tokens ***C*** as described in Equation (4);

  Fine-tune MolT5 with teacher forcing by minimizing the loss in Equation (8);

  Update generator parameters *ψ*;

 **end for**

 **Stage 3: Inference**

 for each input expression profile ***x***_i_ do

  Extract latent conditional embedding ***c***_***i***_;

   Convert ***c***_***i***_ into condition tokens ***C***_*i*_;

   Generate candidate SMILES sequences conditioned on ***C***_*i*_;

   Add generated molecules to ***M***;

 **end for**

 **return *M*;**

## 3. Results

This section evaluates the performance of AET5 from three perspectives. First, we assess whether the transcriptome conditional encoder can effectively capture and preserve informative features from gene expression profiles. Second, we examine whether the MolT5-based molecular generation module can learn intrinsic molecular structural patterns and generate chemically valid, diverse, and distributionally consistent molecules. Finally, we evaluate whether the complete AET5 framework can generate candidate molecules with favorable drug-like, natural-product-like, and pharmacokinetic properties under disease-related transcriptomic conditions. These three evaluations correspond to the key claims of AET5: robust transcriptomic representation learning, reliable conditional molecular generation, and disease-relevant candidate prioritization.

### 3.1. Datasets

To evaluate the generalization capability and effectiveness of AET5 across different disease-related contexts, we used two categories of gene expression profiles: chemical-induced expression profiles and disease-related expression profiles.

**Chemical-induced profiles** were obtained from the LINCS L1000 database [39], which contains transcriptional responses of human cell lines under chemical perturbations. In this study, we used chemical-induced expression signatures as the primary transcriptomic dataset for model training. After preprocessing and filtering, chemical-associated expression profiles with matched compound SMILES representations were retained. These profiles characterize cellular transcriptional responses to diverse molecular perturbations and provide paired transcriptome-molecule data for training the expression-conditioned molecular generation model. The input gene features were based on the 978 landmark genes experimentally measured in the L1000 platform. These genes were used because they are directly assayed and therefore provide reliable expression features. In addition, the original L1000 study demonstrated that these 978 landmark genes can be used to infer the expression of more than 10,000 additional genes with good performance, indicating that they capture representative transcriptomic variation. Thus, they provide a compact and informative feature space for modeling drug-induced transcriptional responses.

**Disease-specific profiles** were collected to evaluate the applicability of AET5 under different disease contexts. For the infectious disease setting, SARS-CoV-2-associated transcriptomic data were obtained from the National Genomics Data Center (NGDC, PRJCA002273) [40] and the Gene Expression Omnibus (GEO, GSE147507) [41]. For the non-infectious disease setting, prostate cancer expression profiles were obtained from the TCGA-PRAD cohort [42]. These disease-related profiles were used as external biological conditions to guide the generation of candidate molecules with potential disease relevance.

For model training and evaluation, the chemical-induced drug perturbation profiles were uniformly divided into training and test sets at a ratio of 9:1. The model was trained only on paired L1000 drug-induced expression profiles and molecular structures. The SARS-CoV-2 and prostate cancer expression profiles were not included in the supervised training set, but were used only as external disease-related conditions in downstream case studies. This design reduces the risk of direct data leakage between model training and disease-specific case-study evaluation.

### 3.2. Baseline

We compared AET5 with several representative gene expression-guided molecular generation models, including two classical generative baselines and two recently proposed models designed for expression-conditioned molecule generation. Since AET5 focuses on SMILES-based molecular sequence generation, SMILES-based LSTM and VAE models were first adopted as classical baselines. Meanwhile, we also recognized the importance of alternative one-dimensional molecular string representations, particularly SELFIES [43], which has been widely explored for improving the robustness and validity of molecular generation. Therefore, in addition to TransGEM [24], which is inherently trained and generated using SELFIES representations, we further implemented SELFIES-based variants of the LSTM and VAE baselines for a more comprehensive comparison. All selected baselines were adapted to conditional *de novo* molecular generation from gene expression profiles and evaluated under the same data split and generation protocol.

**LSTM [**44**]:** LSTM is a classical recurrent neural network architecture widely used for sequence modeling and SMILES-based molecular generation [12]. In this baseline, gene expression profiles are first projected into a low-dimensional condition vector through a linear layer. This condition vector is then used to condition an LSTM-based autoregressive generator for SMILES- and SELFIES-based molecular generation.

**VAE [**45**]:** Variational autoencoders are classical latent-variable generative models that have been widely applied to molecular generation tasks [7]. In this baseline, a conditional VAE is implemented for SMILES- and SELFIES-based molecular generation. Both the encoder and decoder are based on GRU networks [44], and the low-dimensional gene expression condition vector is concatenated with the molecular latent variable before being fed into the decoder.

**Gx2Mol [**25**]:** Gx2Mol is another work by the same authors as GxVAEs for gene expression-guided molecular generation. It first maps gene expression profiles into a low-dimensional latent condition space and then employs an LSTM-based molecular generator to produce SMILES sequences conditioned on the expression-derived representation.

**TransGEM [**24**]:** TransGEM is a Transformer-based conditional molecular generation model. It first compresses gene expression profiles into latent conditional representations and then uses a Transformer decoder to generate SELFIES sequences autoregressively under the guidance of the expression-derived condition.

**GxVAEs [**26**]:** GxVAEs, another work by the same authors as Gx2Mol, employs a dual-VAE architecture for gene expression-guided molecular generation. The first VAE maps gene expression profiles into a low-dimensional latent condition space, while the second VAE generates SMILES molecules conditioned on the expression-derived representation.

**SmilesGEN [**27**]:** SmilesGEN uses a dual-VAE framework for expression-guided molecular generation, consisting of a SMILES VAE for molecule modeling and a profile VAE for gene expression encoding. It further models drug-induced phenotypic effects by constraining the latent space so that post-treatment expression minus molecular representation reconstructs the pre-treatment state, and uses the expression-derived latent code to guide SMILES generation.

### 3.3. Evaluation metrics

To comprehensively evaluate the generated molecules, we grouped the evaluation metrics into two categories: generation quality, structural diversity, and distributional similarity metrics; and drug-likeness, natural-product-likeness, and pharmacokinetic property metrics. The first category includes validity, uniqueness, novelty, internal diversity, SMILES length, and Fréchet ChemNet Distance (FCD), which evaluate whether the model can generate chemically valid, non-redundant, novel, structurally diverse molecules whose distribution is close to that of reference molecules. The second category includes SA, LogP, QED, NP, and ADME-related properties, which assess synthetic feasibility, physicochemical suitability, drug-like character, natural-product-like structural characteristics, and predicted pharmacokinetic behavior. Most molecular properties were computed using RDKit-based cheminformatics pipelines [46], FCD was calculated based on molecular representations extracted by the pretrained ChemNet model [47], while ADME-related properties were predicted using SwissADME [48].

**Validity:** The proportion of generated SMILES strings that can be parsed into chemically valid molecular structures. This metric reflects whether the model can generate syntactically valid and chemically meaningful molecules.

**Uniqueness:** The proportion of non-duplicate molecules among the valid generated molecules. This metric evaluates the model’s ability to avoid repetitive generation.

**Novelty:** The proportion of generated molecules that do not appear in the training set. This metric measures whether the model can generate previously unseen molecular structures rather than simply reproducing training molecules.

**InDiv:** Internal diversity of the generated molecule set, quantified by the average pairwise dissimilarity of molecular fingerprints. This metric reflects the structural diversity and chemical space coverage of the generated molecules.

**FCD:** Fréchet ChemNet Distance, which measures the distributional similarity between generated molecules and reference molecules using ChemNet-based molecular representations. Lower FCD values indicate that the generated molecules are closer to the reference molecular distribution.

**SA [**49**]:** Synthetic accessibility score, which estimates the ease of chemical synthesis. Lower SA scores indicate that molecules are predicted to be easier to synthesize.

**LogP:** The octanol-water partition coefficient, which reflects molecular hydrophobicity and influences physicochemical properties such as membrane permeability and solubility.

**QED [**50**]:** Quantitative estimate of drug-likeness, which summarizes the overall drug-like character of a molecule based on multiple physicochemical and medicinal chemistry properties.

**SMILES length:** The number of characters in the generated SMILES string, used as a coarse descriptor of molecular size and sequence complexity.

**NP [**51**]**: Natural product-likeness score, which estimates the extent to which a molecule resembles natural products based on fragment-level structural patterns. Higher NP scores indicate stronger natural-product-like characteristics, although this score does not directly indicate biological activity.

**ADME-related properties:** Predicted absorption, distribution, metabolism, and excretion characteristics, which reflect the pharmacokinetic behavior of candidate molecules. These properties were evaluated using SwissADME [48].

### 3.4. Evaluation of the transcriptome conditional encoder

To isolate the contribution of the transcriptome conditional encoder from downstream molecular generation, we first evaluated the encoder independently from two complementary perspectives: augmentation robustness and transcriptomic information preservation. Specifically, five augmentation schemes were considered: random dropout, scaling, adaptive Gaussian noise, structural Gaussian noise, and hybrid augmentation. For reconstruction-based evaluation, a three-layer linear network was used to reconstruct the original expression profiles from the compressed representations, and the similarity between the original and reconstructed profiles was used to assess how well the learned representations preserve transcriptomic information.

**Fig 2:**
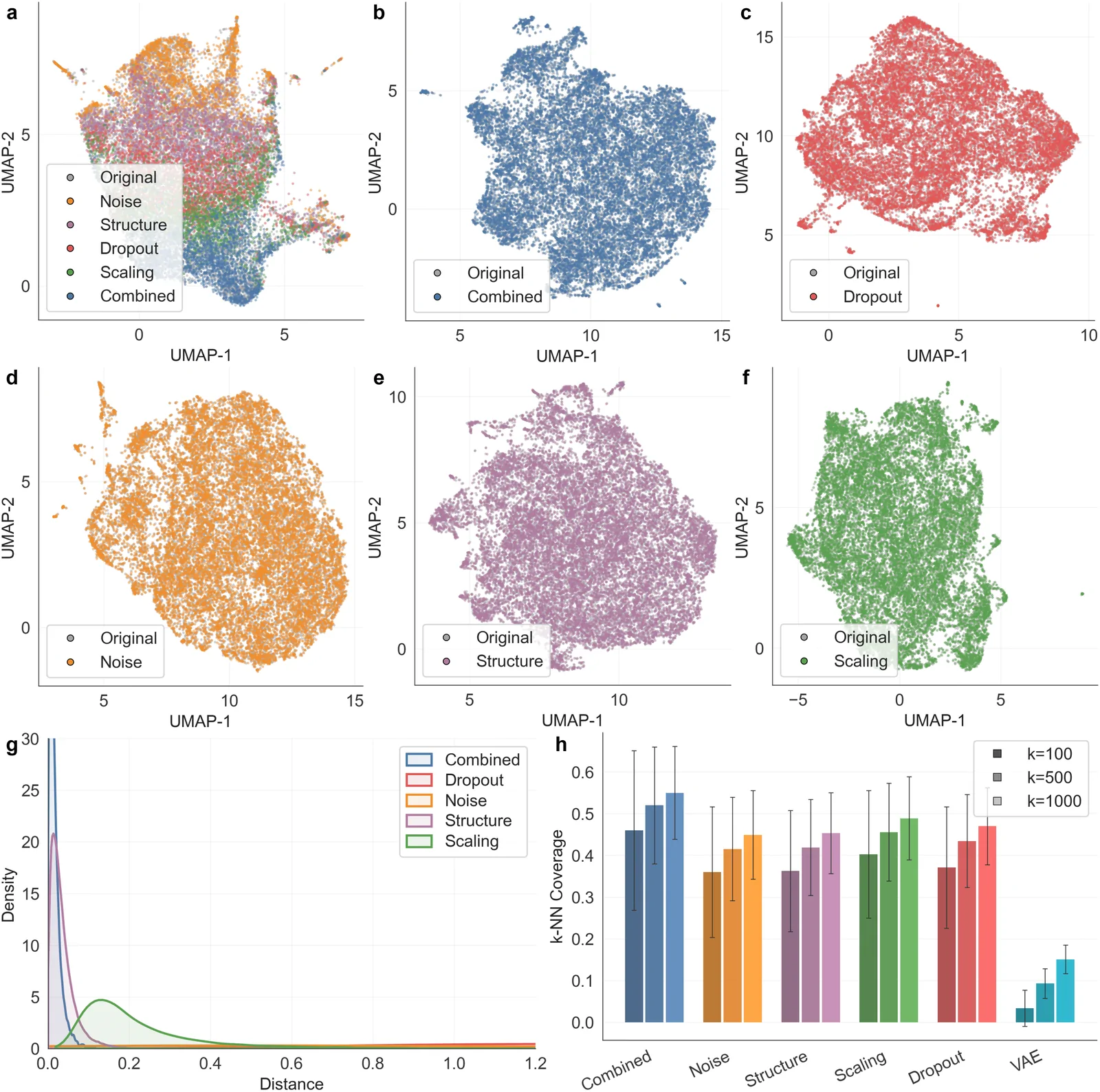
Evaluation of augmentation strategies in the transcriptome conditional encoder (a) UMAP visualization of the original transcriptomic samples and their augmented counterparts under different augmentation strategies. **(b–f)** UMAP visualizations of the encoded representations of the original and augmented samples obtained using encoders trained with hybrid augmentation, random dropout, scaling, adaptive Gaussian noise, and structural Gaussian noise, respectively. **(g)** Kernel density estimation of the distances between encoded original samples and their augmented counterparts under different augmentation strategies. (h**)** *k*-NN coverage of the original samples in the input space under different augmentation strategies, and in the latent space after VAE-based encoding.

As shown in Fig 2, different augmentation strategies produced distinct perturbation patterns in the input space. Fig 2b–2f show the UMAP distributions of the encoded original and augmented samples learned under hybrid augmentation, random dropout, scaling, adaptive Gaussian noise, and structural Gaussian noise, respectively. Compared with the input space, the encoded representations exhibit better alignment between original and augmented samples, indicating that the encoder learns augmentation-invariant features.

We further quantified these effects using the kernel density estimation distributions of distances between encoded original samples and their augmented counterparts, as well as the k-NN coverage in the input space and after VAE-based encoding (Fig 2g and 2h). Smaller and more concentrated distance distributions indicate stronger robustness to augmentation-induced perturbations, whereas higher k-NN coverage reflects better preservation of local neighborhood structure. In Fig 2h, the augmentation-based encoders consistently maintained higher k-NN coverage across different neighborhood sizes, indicating that they better preserved the local structure of the original transcriptomic samples. In contrast, VAE-based encoding resulted in markedly lower k-NN coverage, suggesting a substantial loss of local neighborhood information after compression into the latent space. Among all augmentation strategies, hybrid augmentation achieved the best overall performance, with the closest alignment between original and augmented samples, the most compact distance distribution, and the highest k-NN coverage. Together, the alignment patterns, distance distributions, and k-NN coverage provide complementary evidence that the learned representations are robust to augmentation-induced perturbations while preserving local neighborhood structure more effectively than conventional VAE-based encoding.

**Fig 3:**
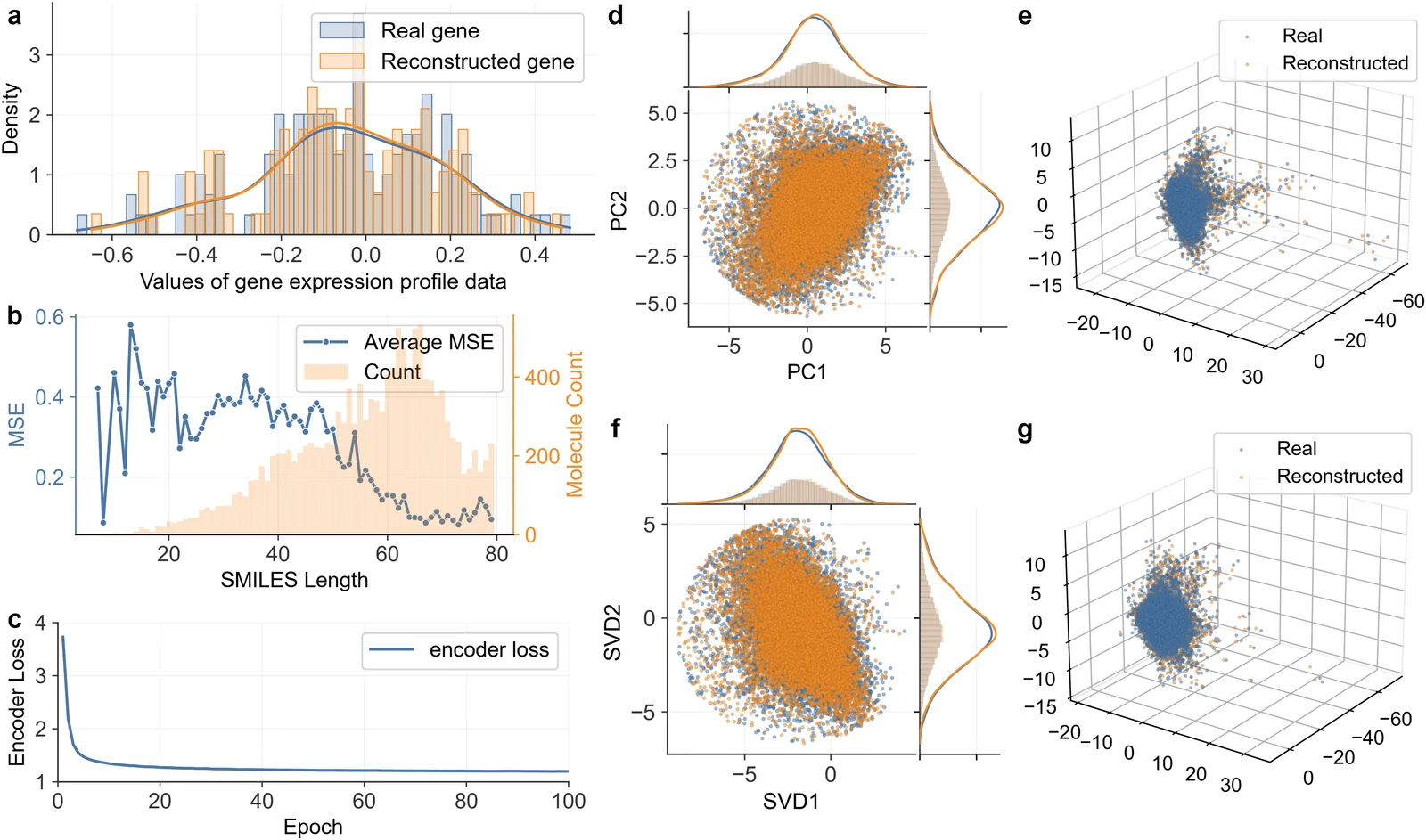
Reconstruction-based evaluation of the Conditional Encoder representations. **(a)** Distributions of expression values before and after reconstruction. **(b)** Reconstruction mean squared error (MSE) across different SMILES lengths, together with the corresponding SMILES length distribution. **(c)** Training loss of the transcriptome conditional encoder. **(d)** Two-dimensional PCA projections of the original and reconstructed expression profiles. **(e)** Three-dimensional PCA projections of the original and reconstructed expression profiles. **(f)** Two-dimensional SVD projections of the original and reconstructed expression profiles. **(g)** Three-dimensional SVD projections of the original and reconstructed expression profiles.

Fig 3a and 3b show the expression value distributions before and after reconstruction, together with the relationship between reconstruction mean squared error (MSE) and SMILES length. The reconstructed profiles were highly similar to the original ones, and the reconstruction error remained low across the major SMILES length range, with larger fluctuations mainly observed in regions containing fewer samples. These results suggest that the transcriptome conditional encoder effectively preserves the major information contained in transcriptomic profiles. In addition, the training loss curve in Fig 3c indicates stable convergence of the reconstruction objective.

As shown in Fig 3d-3g, principal component analysis (PCA) and singular value decomposition (SVD) of the original and reconstructed profiles revealed substantial overlap and highly consistent marginal distributions, further supporting the effectiveness of the learned representations.

### 3.5. Evaluation of molecular generation module

**Fig 4:**
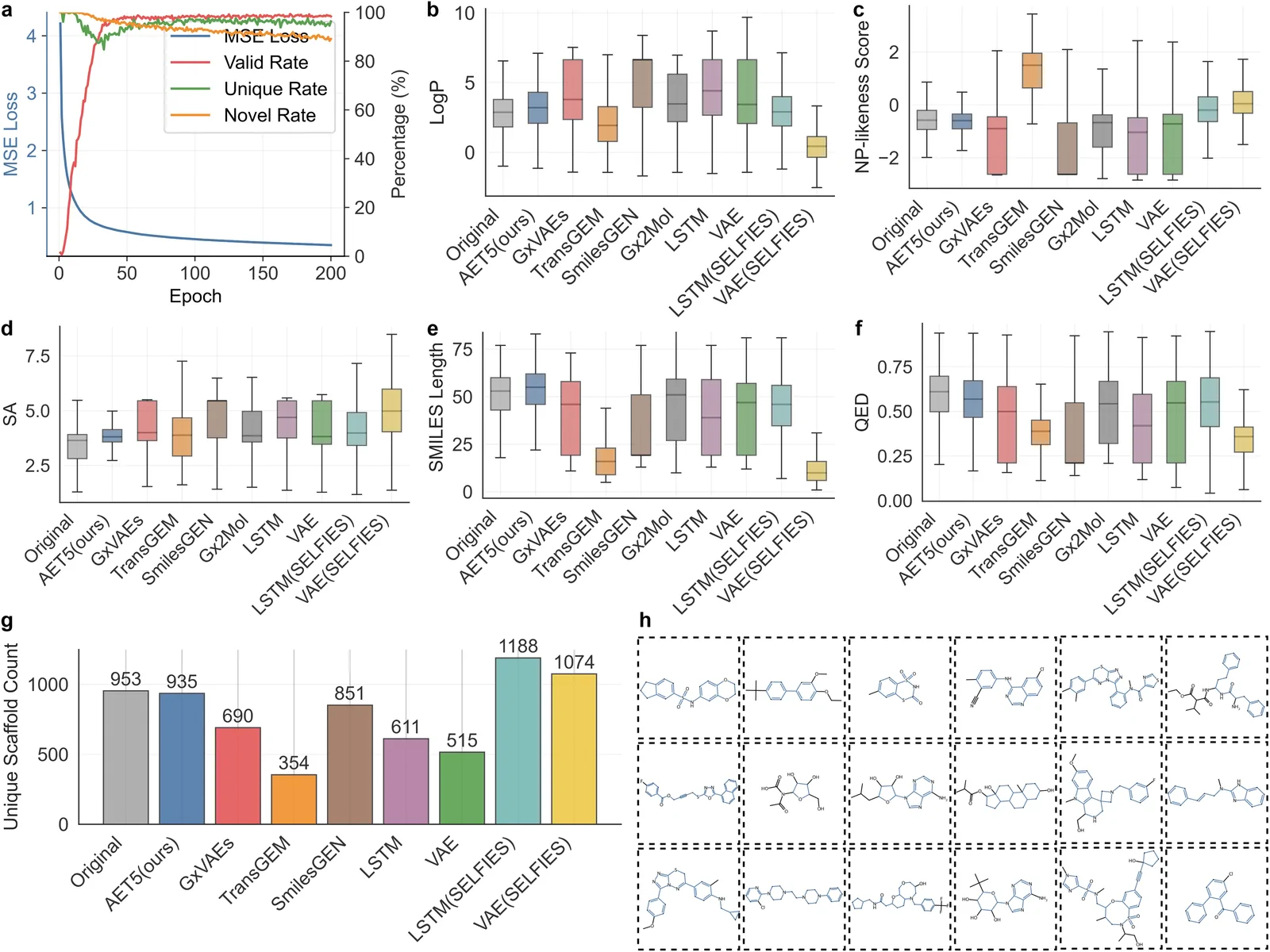
Evaluation and visualization of molecular generation results. **(a)** Training loss and generation metrics during fine-tuning, including MSE loss, validity, uniqueness, and novelty. Violin plots of the generated molecular property distributions for **(b)** LogP, **(c)** NP-likeness score, **(d)** SA, **(e)** SMILES length, and **(f)** QED, compared with the original dataset and baseline models. **(g)** Number of unique scaffolds generated by different models and representative AET5-generated molecules with distinct molecular scaffolds. **(h)** Generated molecules with distinct scaffolds, with scaffolds highlighted in blue.

To evaluate the performance of the fine-tuned MolT5-based molecular generation module, we first compared AET5 with representative baseline models in terms of generation quality, structural diversity, and distributional similarity. The training loss and corresponding evaluation metrics of the generator module are shown in Fig 4a, indicating that the model was effectively optimized during fine-tuning and achieved stable performance improvement.

Table 1 compares AET5 with eight baseline models using validity, uniqueness, novelty, internal diversity, and FCD. Under the same dataset and the same training, validation, and test split, AET5 achieved the most balanced overall performance among the compared methods. Specifically, AET5 obtained 100% validity and 100% uniqueness, indicating that the fine-tuned T5 generator can produce chemically valid molecules while effectively avoiding duplicated outputs. AET5 also achieved the highest novelty score of 97.3%, suggesting that it can generate previously unseen molecules rather than simply reproducing structures from the training set. In terms of internal diversity, AET5 reached 0.87, which was comparable to the highest-performing SELFIES-based models and substantially higher than classical SMILES-based LSTM and VAE baselines.

**Table 1:** Comparative evaluation of eight baselines against AET5.

Model	Validity↑	Uniqueness↑	Novelty↑	InDiv↑	FCD↓
VAE	55.6%	98.8%	97.1%	0.60	11.67
VAE (SELFIES)	**100%**	93.8%	83.2%	0.87	6.69
LSTM	67.7%	91.0%	83.1%	0.59	7.41
LSTM (SELFIES)	**100%**	99.5%	97.2%	**0.88**	11.44
GxVAEs	91.0%	71.4%	36.9%	0.62	6.55
TransGEM (SELFIES)	**100%**	50.3%	85.2%	0.87	63.87
SmilesGEN	94.8%	81.8%	8.9%	0.86	**2.66**
Gx2Mol	74.1%	93.9%	84.2%	0.82	7.63
AET5 (ours)	**100%**	**100%**	**97.3%**	0.87	6.09

Although SmilesGEN achieved the lowest FCD value, its novelty was only 8.9%, suggesting that its generated molecules may be highly similar to the training distribution or affected by overfitting. In contrast, AET5 maintained a competitive FCD value while preserving high validity, uniqueness, novelty, and internal diversity. Compared with Gx2Mol, AET5 achieved higher validity, uniqueness, novelty, and internal diversity, while also obtaining a lower FCD value, indicating better overall generation quality and stronger distributional consistency with the reference set. In addition, the 100% validity observed in several SELFIES-based models is partly attributable to the inherent robustness of the SELFIES representation. Therefore, validity should be interpreted together with uniqueness, novelty, diversity, and FCD when comparing different molecular representations.

We further examined whether the generated molecules preserved the physicochemical and drug-likeness-related property distributions of the reference molecules. Table 2 summarizes the mean and standard deviation of SMILES length, QED, LogP, SA, and NP for AET5 and the baseline models, whereas Fig 4b-4f shows the corresponding boxplots. Importantly, the statistics in Table 2 were computed from valid generated molecules without penalization, while the boxplots were based on penalized distributions that incorporated invalid or non-novel molecules to provide a stricter assessment of generation quality. Together, these analyses enable evaluation of both central tendency and overall distributional behavior, including medians, interquartile ranges, dispersion, and the impact of low-quality generations.

**Table 2:** Mean and standard deviation of AET5 and baseline models for the second-category metrics.

Model	Length	QED	LogP	SA	NP
Original	49.78 ± 12.86	0.60 ± 0.16	2.79 ± 1.59	3.40 ± 0.83	-0.50 ± 0.88
AET5 (ours)	52.43 ± 12.12	0.60 ± 0.14	3.25 ± 1.38	3.32 ± 0.91	-0.72 ± 0.63
GxVAEs	52.89 ± 11.75	0.60 ± 0.14	2.77 ± 1.44	3.64 ± 0.75	-0.54 ± 0.56
Gx2Mol	55.23 ± 10.29	0.58 ± 0.13	3.02 ± 1.39	3.74 ± 0.67	-0.56 ± 0.46
TransGEM	21.60 ± 9.59	0.38 ± 0.13	2.72 ± 1.78	4.52 ± 1.25	1.40 ± 0.69
SmilesGEN	49.43 ± 13.14	0.60 ± 0.15	2.97 ± 1.56	3.39 ± 0.84	-0.49 ± 0.92
LSTM	56.59 ± 9.88	0.51 ± 0.16	3.47 ± 1.67	3.82 ± 0.68	-0.51 ± 0.60
VAE	68.26 ± 20.63	0.29 ± 0.25	8.64 ± 6.16	4.11 ± 0.94	-0.34 ± 0.54
LSTM (SELFIES)	45.86 ± 13.61	0.56 ± 0.19	2.95 ± 1.50	4.15 ± 1.15	-0.11 ± 0.74
VAE (SELFIES)	56.66 ± 16.00	0.48 ± 0.21	3.76 ± 2.14	4.40 ± 1.18	-0.22 ± 0.72

As shown in Table 2 and Fig 4b-4f, AET5 closely matched the original molecular distribution across multiple properties. For SMILES length, AET5 generated molecules with a mean length of 52.43, which was close to the original mean of 49.78 and showed a similar level of dispersion. For QED, AET5 achieved the same mean value as the original dataset, 0.60, indicating that the generated molecules retained a comparable level of drug-likeness. For SA, AET5 obtained a mean score of 3.32, close to the original value of 3.40, suggesting that the generated molecules remained synthetically accessible. For NP, AET5 showed a mean score of -0.72 compared with -0.50 for the original dataset, indicating a moderate shift toward less natural-product-like structures, although the overall distribution remained within a comparable range. Although the LogP distribution of AET5 was slightly shifted toward higher values compared with the original dataset, it remained within a reasonable range and was substantially less distorted than that of VAE, which showed an excessive LogP mean and large variance.

By contrast, several baseline models exhibited clear deviations from the original distribution. TransGEM generated substantially shorter molecules, with a mean SMILES length of 21.60, and showed lower QED, higher SA scores, and a markedly shifted NP distribution, suggesting that its generated molecules were structurally simpler and less aligned with the reference chemical space. VAE produced molecules with excessively long SMILES strings and markedly inflated LogP values, indicating unstable property distributions. LSTM also showed higher molecular length and LogP values than the original dataset. Gx2Mol showed relatively reasonable property distributions, but its generated molecules were slightly longer, had lower QED, and exhibited higher SA scores than those of the original dataset and AET5, indicating a moderate deviation in drug-likeness and synthetic accessibility. Overall, AET5 better preserved the property distribution trends of the reference molecules across SMILES length, QED, LogP, SA, and NP, including peak locations, concentration regions, and dispersion patterns.

We also evaluated scaffold diversity by counting the number of unique scaffolds generated by each model. As shown in Fig 4g, AET5 generated 960 unique scaffolds, closely matching the original dataset with 953 scaffolds and exceeding most baseline models, including Gx2Mol. To further provide a structural-level visualization of this diversity, Fig 4h presents representative AET5-generated molecules with their extracted molecular scaffolds highlighted in blue. These examples show that AET5 can generate molecules containing diverse core scaffolds and substituent patterns, supporting its ability to explore heterogeneous chemical structures under transcriptomic conditions. Although LSTM-based SELFIES generated a larger number of unique scaffolds, scaffold count alone does not guarantee favorable molecular quality and should be interpreted together with validity, novelty, internal diversity, FCD, and property distributions. These results suggest that AET5 can generate structurally diverse molecules while maintaining a property profile close to that of the reference molecules.

To further investigate the contribution of each component in the fine-tuned T5 generation module, we conducted ablation experiments. As shown in Table 3, all ablated variants performed worse than the complete AET5 model, demonstrating that the final performance improvement was not caused by a single isolated component but by the combined effect of the proposed design choices.

**Table 3:** Comparative evaluation of ablated variants against AET5.

Model	Validity↑	Uniqueness↑	Novelty↑	InDiv↑	FCD↓
w/o embedding	94.4%	71.2%	72.7%	0.78	13.97
w/o pretraining	89.5%	65.0%	48.1%	0.83	10.65
Dropout only	95.6%	71.7%	73.4%	0.77	12.95
Adaptive Gauss only	94.8%	72.2%	72.8%	0.78	14.14
Structured Gauss only	97.7%	57.8%	62.3%	0.69	27.73
Scaling only	96.0%	74.0%	74.7%	0.77	15.59
AET5 (ours)	**100%**	**100%**	**97.3%**	**0.87**	**6.09**

Removing the embedding component led to clear degradation across all metrics, with validity decreasing from 100% to 94.4%, uniqueness from 100% to 71.2%, and novelty from 97.3% to 72.7%. This result suggests that the embedding design is important for effectively integrating conditional information and guiding molecular generation. Removing pretrained initialization caused an even stronger decline, especially in novelty, which dropped to 48.1%. This indicates that pretrained molecular sequence knowledge is critical for learning valid molecular syntax, structural regularities, and generalizable chemical patterns.

The variants using only one regularization or perturbation strategy also showed limited performance. The “Dropout only”, “Adaptive gauss only”, and “Scaling only” variants achieved relatively high validity but suffered from substantially reduced uniqueness and novelty, indicating that these individual strategies alone were insufficient to support diverse and novel molecule generation. The “Structured gauss only” variant achieved 97.7% validity but showed the lowest uniqueness among the ablated models and the worst FCD value, suggesting that using structured Gaussian perturbation alone may restrict the generated chemical space or introduce distributional mismatch.

In contrast, the complete AET5 model achieved 100% validity, 100% uniqueness, 97.3% novelty, 0.870 internal diversity, and the lowest FCD among all ablated variants. These results demonstrate that the proposed components work synergistically to improve molecular validity, reduce duplication, enhance novelty, maintain structural diversity, and better align the generated molecules with the reference chemical distribution.

**Fig 5:**
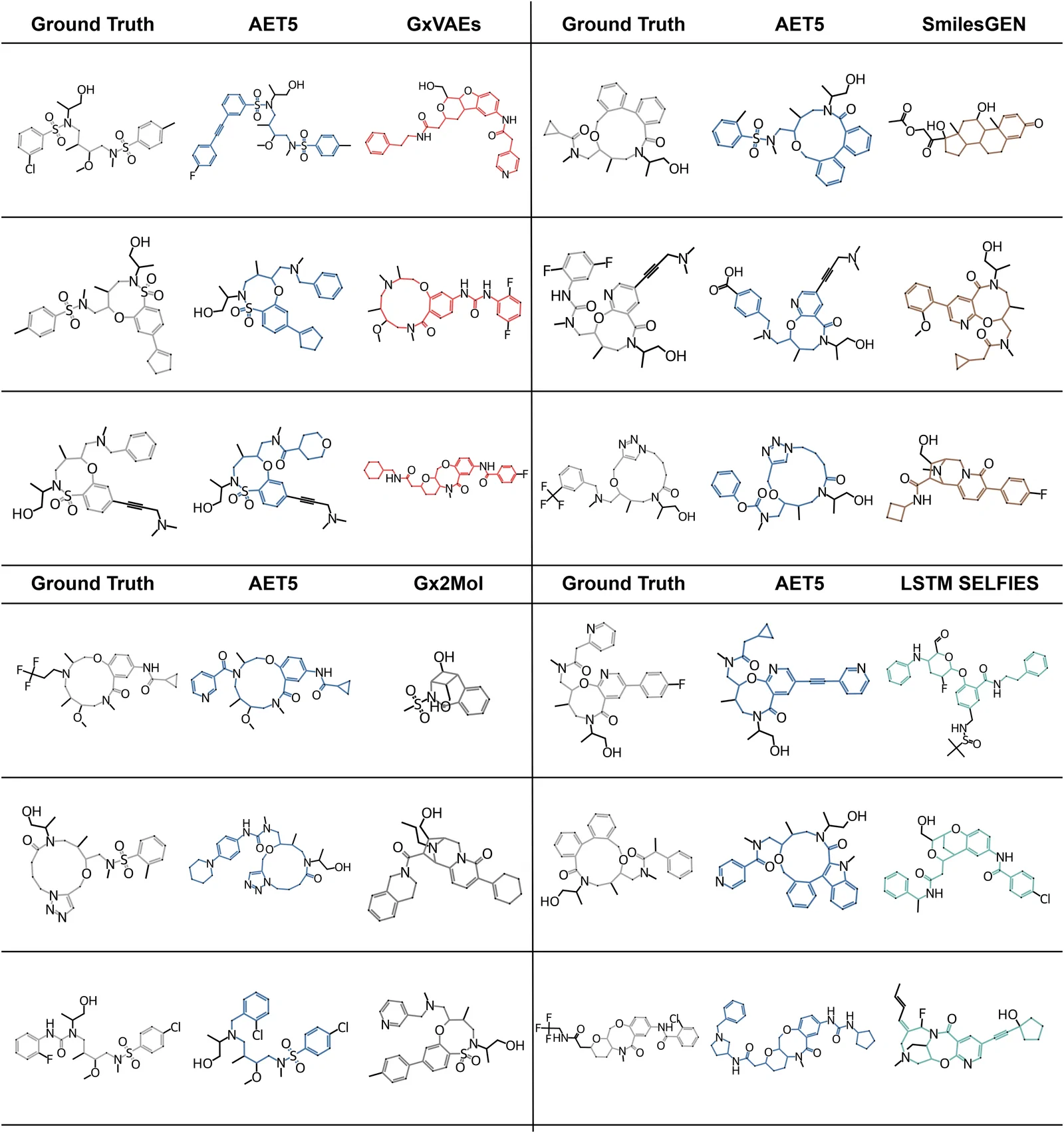
Qualitative comparison of molecules generated by AET5 and baseline models under the same transcriptomic conditions.

To further illustrate the structural relevance of the generated molecules at the individual-sample level, we visualized representative examples by comparing the ground-truth molecules with the corresponding molecules generated by AET5 and selected baseline models under the same transcriptomic conditions (Fig 5). Across these examples, AET5-generated molecules showed closer structural correspondence to the ground-truth molecules, preserving key scaffold-level features, ring systems, linker patterns, and functional group arrangements. In contrast, the baseline models often produced molecules with larger deviations in scaffold topology or substructure organization, although some local chemical motifs were partially retained. These qualitative comparisons provide additional evidence that AET5 can translate transcriptomic conditional information into chemically meaningful molecular structures while maintaining structural diversity rather than simply reproducing the reference molecules.

Overall, these comparative, qualitative, and ablation results show that the fine-tuned T5-based molecular generation module can effectively leverage transcriptomic conditional information to generate valid, non-redundant, novel, and structurally diverse molecules. Compared with baseline models, AET5 provides a more favorable balance between novelty, diversity, structural relevance, and distributional consistency, while the ablation study confirms the necessity of its key components for stable and high-quality conditional molecular generation.

### 3.6. Case study

To further assess the practical utility of AET5 in real-world disease settings, we conducted two computational case studies in mechanistically distinct scenarios: SARS-CoV-2 infection and PC. These analyses were designed to evaluate three aspects of the proposed framework. First, we examined whether AET5 remains applicable under noisy and heterogeneous transcriptomic conditions, which is important for assessing the robustness of the decoupled architecture and the parameter-efficient fine-tuning strategy. Second, we investigated whether the learned disease-conditioned signals can be associated with biologically meaningful loci and candidate targets, thereby providing supportive evidence that the conditional representations may capture disease-related information rather than merely serving as abstract control variables. Third, we tested whether a single expression-conditioned generative framework can be applied across distinct disease contexts and generate candidate molecules with favorable predicted pharmacokinetic properties and plausible target-binding potential.

**Fig 6:**
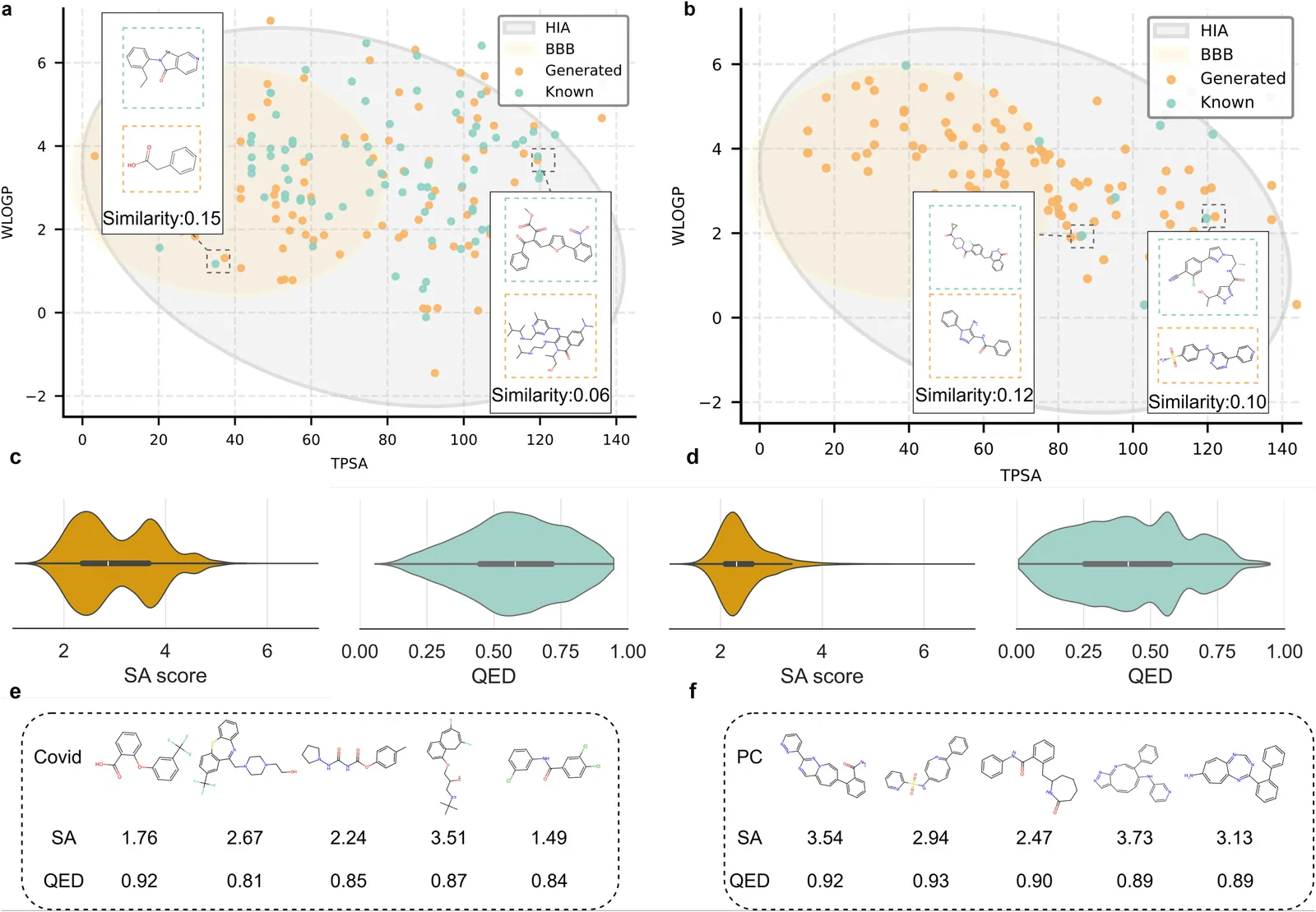
ADME property analysis and representative molecules generated under disease-specific conditions. **(a, b)** TPSA–WLOGP distributions of generated molecules and known active compounds under SARS-CoV-2 and PC conditions, respectively, projected onto the BOILED-Egg model. Representative generated-known molecule pairs with low structural similarity are shown. **(c, d)** QED and SA score distributions of valid and non-redundant generated molecules under SARS-CoV-2 and PC conditions, respectively. **(e, f)** Representative generated molecules selected under SARS-CoV-2 and PC conditions, respectively, with their predicted SA and QED values.

For the first scenario, we considered SARS-CoV-2 as a representative example of a rapidly emerging infectious disease, where transcriptomic data are often limited, noisy, and collected from heterogeneous sources. The dataset included collective samples from the National Genomics Data Center and individual-level samples from the National Center for Biotechnology Information, which exhibit substantial differences in expression patterns. To preserve biologically relevant information from both sources while encouraging conditional exploration, we adopted a mixed-sample strategy to construct multiple potential expression profiles for molecular generation. Under each disease-specific condition, AET5 generated candidate molecules, which were first subjected to RDKit-based validity checking and canonical SMILES-based deduplication to remove invalid and redundant structures. The remaining valid and non-redundant molecules were further filtered according to predicted synthetic accessibility, retaining candidates with SA score < 4.0 for downstream evaluation. To preliminarily assess their biological relevance, we used the DeepCE model [29], to predict the transcriptomic perturbation profile induced by each retained molecule and calculated its correlation with the corresponding disease-specific expression signature. For each disease condition, molecules showing the strongest negative correlations with the disease-specific profile were selected as prioritized candidates for subsequent docking-based target-binding evaluation and molecular dynamics simulations. The ADME-related properties and drug-likeness of these prioritized candidates were then analyzed using the SwissADME platform and QED estimation. As shown in Fig 6a, 6c, most SARS-CoV-2-conditioned molecules fall within or near the absorbable region of the BOILED-Egg model [52], In addition, the majority of generated molecules exhibit SA scores below 4 and QED values above 0.5, indicating plausible pharmacokinetic properties, favorable predicted synthetic accessibility, and drug-likeness. Representative generated molecules are also shown in Fig 6e together with their SA and QED values, further supporting their favorable predicted synthetic accessibility and drug-likeness. Notably, several generated molecules are located close to known compounds in the TPSA–WLOGP space while exhibiting low structural similarity, suggesting that AET5 can generate novel candidates that preserve desirable pharmacokinetic characteristics without simply reproducing known structures. Taken together, these results suggest that AET5 remains applicable under noisy and heterogeneous transcriptomic conditions, supporting the potential robustness of the proposed framework in small-sample and rapidly evolving disease scenarios.

For the second scenario, we selected PC as a representative example of a disease with relatively abundant data but complex pathological mechanisms. In this setting, the key challenge is not only to generate chemically plausible molecules, but also to explore whether disease-related biological signals and potential therapeutic targets can be associated with the generation process. To this end, we extracted attention scores from the molecular generator to quantify the contribution of individual gene loci to token-level molecular decisions. These loci were then cross-referenced with the Open Targets database [53] to identify known proteins associated with high-confidence model-attended genes. Among the 978 loci used in the model, 25 were matched to PC-related targets with confidence scores greater than 0.5, suggesting potential disease relevance. The top 10 loci ranked by attention score are listed in S1 Table. The observed overlap between highly attended loci and known disease-associated targets provides supportive evidence that the disease-conditioned signals learned by AET5 may capture biologically relevant information and may help guide molecular generation toward disease-related cellular mechanisms, rather than acting solely as statistical prompts.

To further assess the potential biological relevance of these loci, we selected targets from the top 50 attention-ranked genes that also had complete structural information, known binding pockets, and available reference molecules for downstream evaluation. Specifically, *CHEK2* (PDB ID: 2YCF) and *TUBB6* (PDB ID: 6AB2) were chosen as candidate protein targets. *CHEK2* [54] is a checkpoint kinase involved in DNA damage response and cell-cycle regulation, and it plays an important role in the proliferation and repair mechanisms of PC cells. *TUBB6* [55], a member of the tubulin family, participates in cytoskeletal assembly and dynamics and is closely related to tumor cell migration and division. These proteins were therefore used as biologically plausible targets for subsequent docking-based assessment. In parallel, ADME analysis was also performed for PC-conditioned generated molecules in comparison with known compounds. As shown in Fig 6b, 6d, and 6f, the generated molecules occupy a pharmacokinetic space similar to that of known compounds in the BOILED-Egg model. In Fig 6d, the PC-conditioned molecules are mainly distributed in a low SA-score range, with most candidates showing SA scores below 4, indicating favorable predicted synthetic accessibility. In contrast, their QED values display a relatively broad distribution around 0.5, with candidates present on both sides of this threshold, suggesting variable but overall plausible drug-likeness. Representative candidates with favorable predicted SA and QED values are further shown in Fig 6f, suggesting that the model can generate chemically plausible and pharmacologically relevant candidates under cancer-related expression conditions.

In addition to the two PC-related targets, two key SARS-CoV-2 proteins, *Nsp3* [56] (PDB ID: 7LMD) and RNA-dependent RNA polymerase [57] (*RdRp*, PDB ID: 9I1S), were selected for further validation. *Nsp3* is involved in viral polyprotein processing and immune evasion, whereas *RdRp* is essential for viral RNA replication, making both proteins important antiviral targets. The generated candidate molecules were evaluated by molecular docking using AutoDock Vina [58] and by molecular dynamics simulations using AmberTools20 [59].

**Fig 7:**
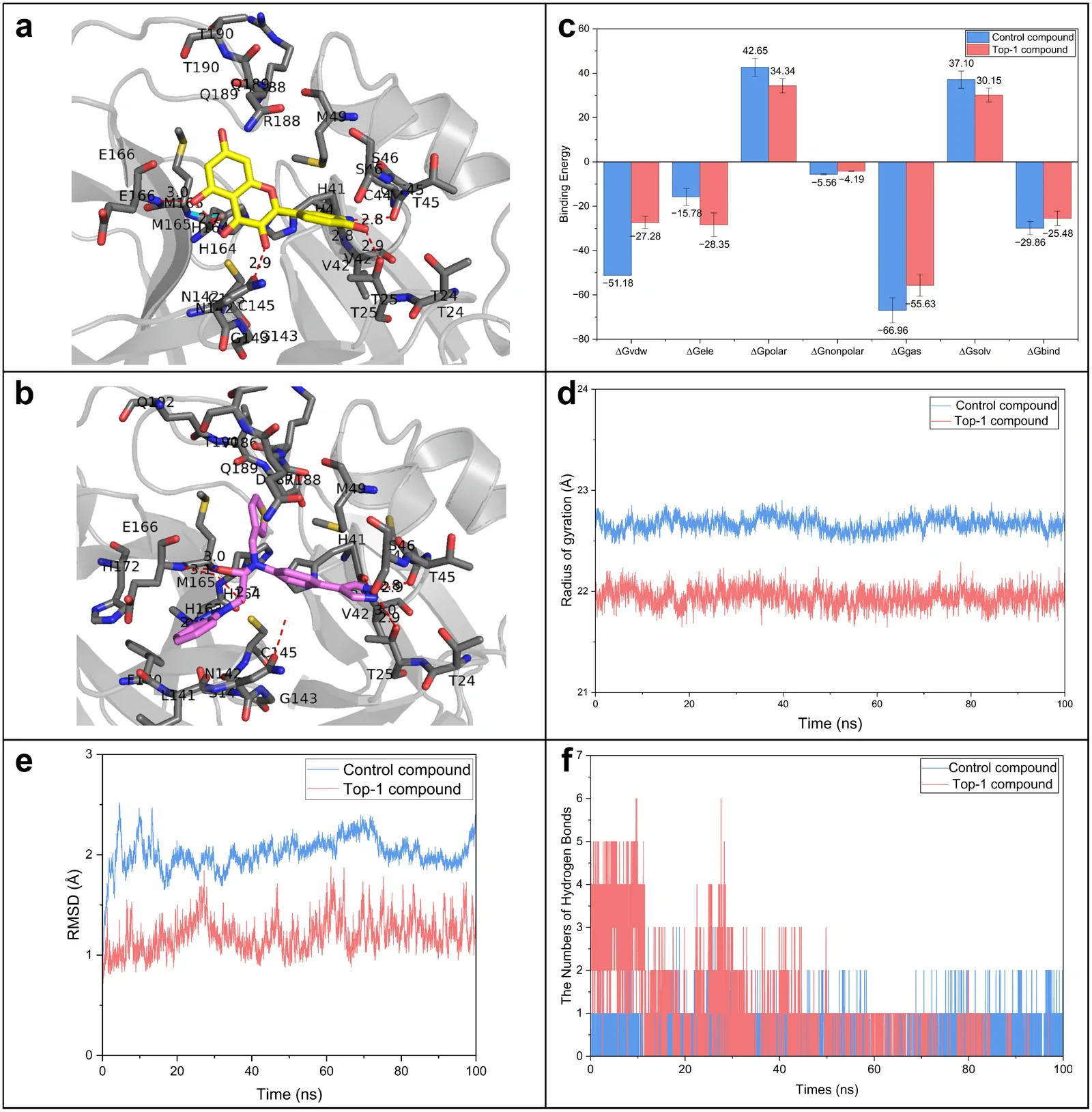
Comparison of ligand–protein binding between AET5-generated and control molecules for the 7LMD SARS-CoV-2 target. **(a)** 3D interaction and binding site of the generated molecule in the ligand–protein complex. **(b)** 3D interaction and binding site of the control molecule in the ligand–protein complex. **(c)** Comparison of binding energies. **(d)** Time-dependent radius of gyration. **(e)** Time-dependent number of hydrogen bonds. **(f)** Time-dependent root mean square deviation (RMSD).

Fig 7 shows the binding behavior of the generated molecules and control molecules for the SARS-CoV-2 target 7LMD. Fig 7a and 7b present the 3D binding conformations, showing that AET5 can generate novel molecules that occupy a binding pocket similar to that of the control compound while maintaining clear structural differences. The binding energy comparison in Fig 7c indicates that the generated molecule exhibits a more favorable predicted binding profile than the control molecule. In addition, the time-dependent radius of gyration, hydrogen-bond number, and RMSD curves in Fig 7d-7f suggest that the generated ligand–protein complex maintains competitive conformational compactness and dynamic stability throughout the simulation.

**Fig 8:**
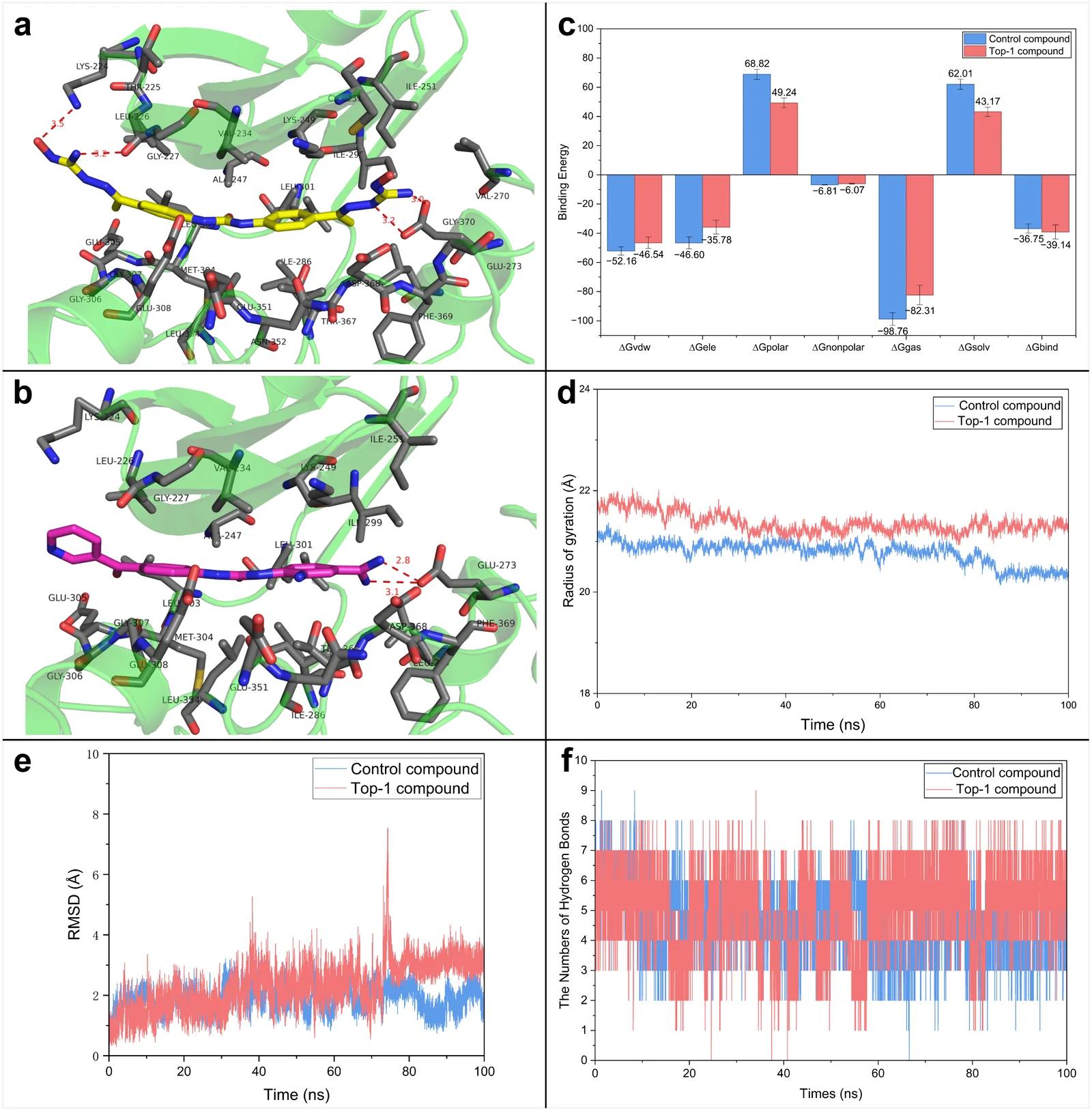
Comparison of ligand–protein binding between AET5-generated and control molecules for the 2YCF PC target. **(a)** 3D interaction and binding site of the generated molecule in the ligand–protein complex. **(b)** 3D interaction and binding site of the control molecule in the ligand–protein complex. **(c)** Comparison of binding energies. **(d)** Time-dependent radius of gyration. **(e)** Time-dependent number of hydrogen bonds. **(f)** Time-dependent RMSD.

Similar analyses were conducted for additional targets, including 2YCF, 6AB2, and 9I1S, with the corresponding results shown in Fig 8, S3 and S4 Figs Across these targets, the generated molecules showed favorable or comparable predicted binding energies and stable interaction patterns relative to the control compounds, although the detailed dynamic behaviors varied across targets. These results suggest that AET5 does not merely generate chemically valid structures, but can also produce candidate molecules with plausible computational target-binding potential in both infectious disease and cancer settings.

Overall, the two case studies provide complementary computational evidence for the main design goals of AET5. The SARS-CoV-2 results suggest that the framework can remain applicable under limited, noisy, and heterogeneous transcriptomic inputs, supporting the potential robustness of the decoupled architecture and parameter-efficient generator adaptation. The PC results show that the learned disease-conditioned signals can be associated with biologically meaningful loci and known disease-associated targets, providing supportive evidence for the biological relevance of the proposed conditional representation learning strategy. Across both viral infection and cancer scenarios, AET5 generated candidate molecules with favorable predicted pharmacokinetic properties and plausible target-binding behavior in docking and molecular dynamics analyses. These findings highlight the potential generality and practical utility of the proposed expression-conditioned molecular generation framework, while further biochemical or cellular experiments would be required to confirm the therapeutic activity of the generated candidates.

## 4. Discussion

In this study, we developed AET5, a transcriptome-guided molecular generation framework that combines contrastive transcriptomic representation learning with a pre-trained sequence-to-sequence molecular generator. By conditioning molecular generation on disease-reversal expression profiles, AET5 links disease-associated transcriptional signals with de novo chemical design. The results show that AET5 achieves a favorable balance among validity, uniqueness, novelty, internal diversity, scaffold diversity, and distributional consistency, suggesting that transcriptomic profiles can provide useful conditional signals for generating biologically relevant molecules.

**Fig 9:**
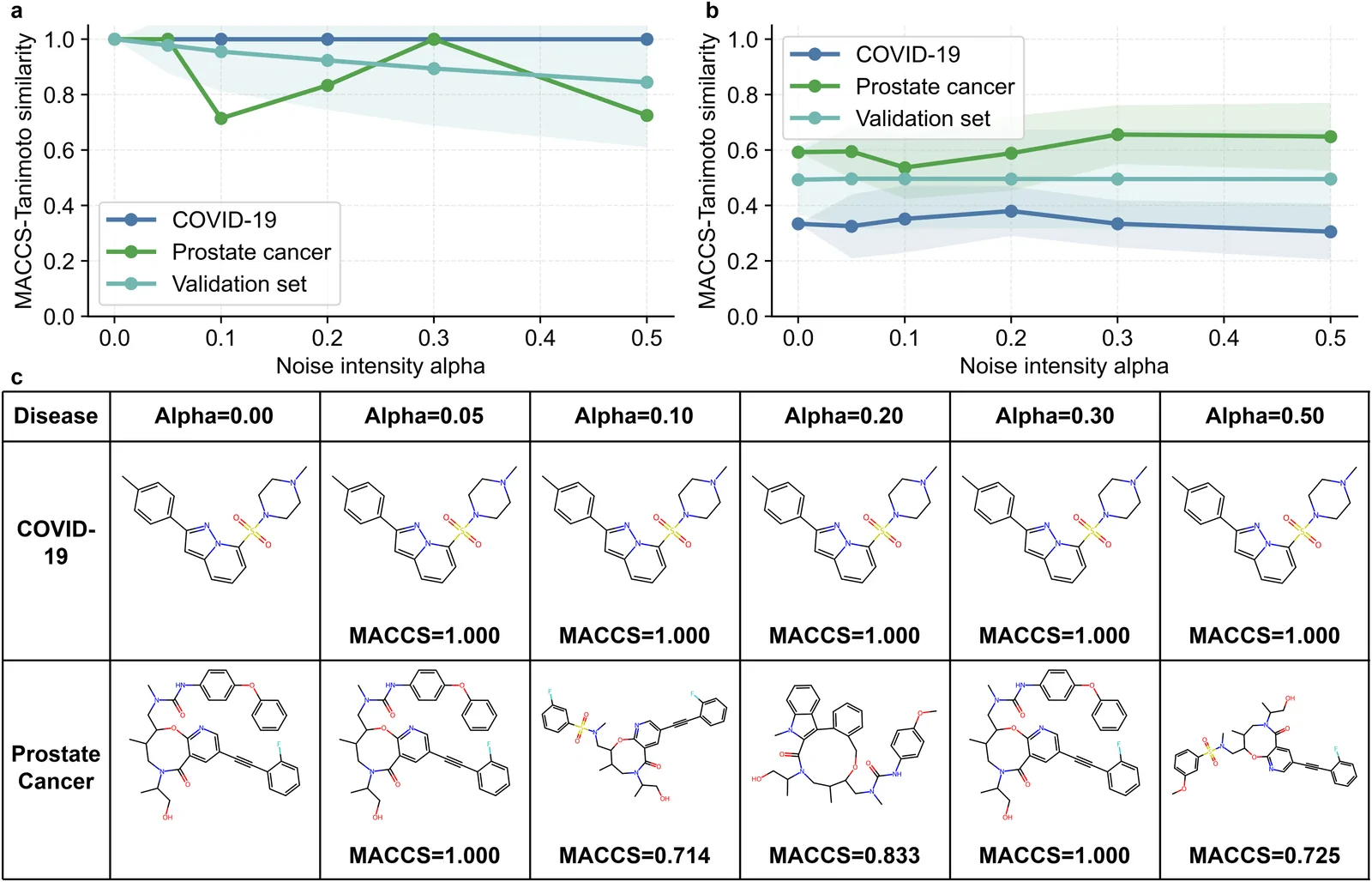
Robustness analysis of AET5 under different noise intensities. **(a)** Maximum similarity. **(b)** Average similarity. **(c)** Molecules generated under greedy decoding for COVID-19 and PC across noise intensities.

A key challenge in transcriptome-guided molecular generation is the noise inherent in disease expression profiles. Noisy disease-reversal profiles may still generate chemically valid molecules, but these molecules may become less related to the intended disease state. To evaluate this issue, we performed a noise-control analysis by adding random noise with different intensities to disease-reversal expression profiles and comparing the generated molecules with those produced from the original profiles. As shown in Fig 9, AET5 generally retained stable molecular generation under low-to-moderate noise, but the observed robustness depended on the decoding strategy and disease context. Under greedy decoding (Fig 9a), the COVID-19 case showed particularly stable MACCS-Tanimoto similarity across all tested noise intensities, indicating that the top-ranked generated molecule was unchanged after transcriptomic perturbation. In contrast, the prostate cancer case displayed larger fluctuations, suggesting higher sensitivity to input noise. Under sampling-based generation (Fig 9b), the similarity at noise intensity α = 0 represents the inter-group similarity among molecules generated under the same original condition, reflecting the baseline diversity of the generator rather than noise-induced changes. Across increasing noise intensities, the average similarity did not show an abrupt collapse, indicating that AET5 maintained a relatively stable generated chemical distribution under moderate perturbation. Representative greedy-decoded molecules for COVID-19 and prostate cancer are shown in Fig 9c, further illustrating the disease-specific differences in robustness. These results indicate that transcriptomic noise can affect the generated chemical space, but AET5 does not collapse abruptly under moderate perturbation.

The robustness of AET5 may be partly attributed to its transcriptomic conditional encoder and augmentation strategy. As shown in Fig 2, different augmentation strategies produced distinct latent distributions because they perturb transcriptomic profiles in different ways. Random dropout removes part of the gene-level signal, which may improve tolerance to missing or weakly measured genes but can also disturb disease-relevant expression patterns. Scaling changes the global magnitude of expression profiles while largely preserving expression direction. Adaptive Gaussian noise introduces gene-wise perturbations according to expression variability, which may help reduce sensitivity to individual noisy genes. Structural Gaussian noise incorporates gene-dependency information and may help preserve co-expression relationships and pathway-level correlations. The hybrid strategy combines these perturbations and provides relatively broad but controlled coverage of the transcriptomic space, as suggested by the UMAP patterns, distance distributions, and k-NN coverage results in Fig 2h.

**Fig 10:**
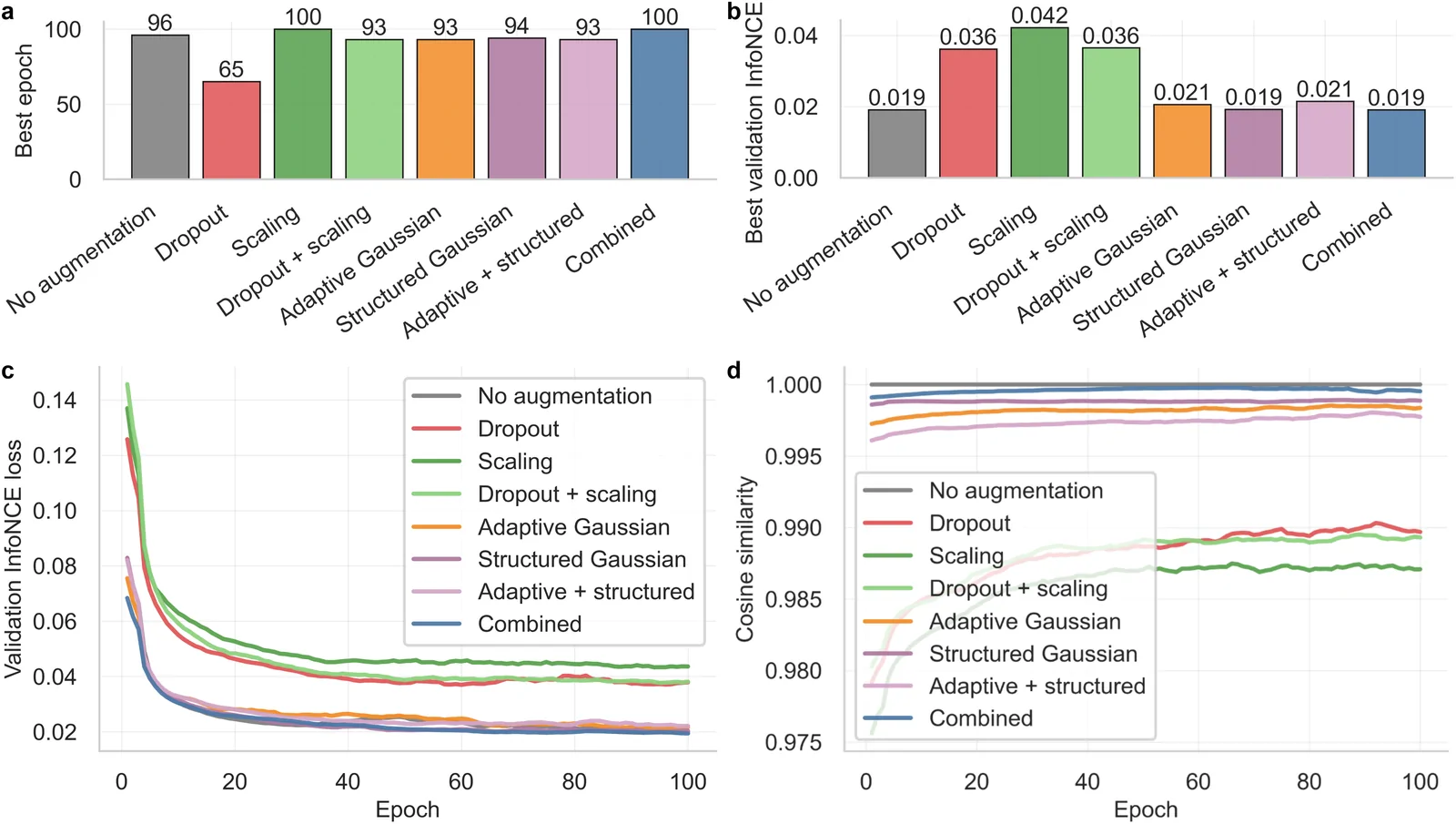
Effects of augmentation strategies on encoder training. **(a)** Best-validation epoch. **(b)** Best validation InfoNCE loss. **(c)** Validation InfoNCE loss over epochs. **(d)** Embedding cosine similarity over epochs.

Fig 10 further compares the training behavior under different augmentation settings. Fig 10a shows the epoch at which the best validation InfoNCE loss was reached, providing an indication of convergence speed, while Fig 10b shows the corresponding best validation loss. Fig 10c shows that dropout, scaling, and dropout with scaling tended to converge to higher validation losses, whereas Gaussian-noise-based strategies, especially the combined strategy, maintained lower validation losses during later epochs. Fig 10d shows that dropout- and scaling-based strategies had lower cosine similarity between embeddings of augmented samples from the same original sample, while adaptive Gaussian noise, structural Gaussian noise, and the combined strategy showed higher embedding consistency. These results suggest that Gaussian-noise-based augmentation may improve representation stability without clearly impairing convergence.

The ablation analysis in Table 3 provides further insight into why the full AET5 framework achieved a better balance across multiple generation metrics. The comparison between the full model and the two module-ablated variants suggests that both transcriptomic embedding and molecular generator pretraining are important for effective transcriptome-guided molecular generation. When the transcriptomic embedding module was removed, the generated molecules still showed relatively high validity, but uniqueness, novelty, internal diversity, and FCD were all less favorable than those of the full model. This indicates that transcriptomic representation learning is not only a conditioning step but also contributes to organizing the input disease-reversal signals into a latent space that supports more diverse and distributionally consistent molecular generation. Similarly, removing molecular generator pretraining led to a marked reduction in novelty and overall generation quality, suggesting that pretraining provides essential chemical sequence knowledge that helps the model explore chemically meaningful structures. Therefore, the superior performance of AET5 is likely derived from the complementary roles of these two components: transcriptomic embedding provides biologically informed conditional signals, whereas molecular pretraining provides chemically informed generative capacity.

The same ablation analysis also supports the benefit of hybrid augmentation for conditional molecular generation. Although several single-augmentation variants maintained relatively high validity, they did not achieve the same balance among uniqueness, novelty, internal diversity, and FCD as the full AET5 model. In particular, the structured Gaussian-only setting showed reduced uniqueness, novelty, and internal diversity, together with a higher FCD, suggesting that structural perturbation alone may be insufficient or overly restrictive when not balanced by other augmentations. Overall, these results support the use of a hybrid augmentation strategy together with contrastive transcriptomic representation learning and a pre-trained molecular generator.

We further performed a one-factor-at-a-time hyperparameter sensitivity analysis to guide the final configuration of AET5 (S5 Fig). Because exhaustive grid search over noise parameters, contrastive temperature, and decoding strategies is computationally prohibitive, we varied one factor at a time while keeping the others fixed and evaluated the resulting trends using encoder loss, generator training and validation losses, molecular validity, internal diversity, SA score, and QED. The tested ranges of noise probability and noise strength showed relatively stable loss behavior, whereas the contrastive temperature mainly affected the encoder contrastive learning loss. Different top-k and top-p decoding settings showed trade-offs among validity, diversity, and molecular quality metrics. Therefore, the final hyperparameters were empirically selected rather than claimed to be globally optimal: we first identified promising ranges from the single-factor analyses, then evaluated several candidate combinations, and finally used the configuration with the best overall balance in the main experiments.

Several limitations remain. First, the current validation of AET5 is mainly computational, including molecular similarity analysis, ADME prediction, docking, and molecular dynamics simulations. These results support the chemical plausibility of generated molecules but cannot directly confirm therapeutic activity, target selectivity, toxicity, or pharmacokinetic safety. Experimental validation using biochemical assays, cell-based models, and in vivo studies is still required. Second, although AET5 showed robustness to low-to-moderate transcriptomic noise in our analyses, its performance may still depend on the quality of disease-reversal expression profiles. Highly noisy, systematically biased, or incomplete transcriptomic inputs could weaken disease-relevant signals and guide the model toward chemically valid but biologically less relevant molecules. Third, the current framework does not explicitly incorporate target structures, drug-target interactions, pathway annotations, or protein-protein interaction networks, which limits mechanistic control over molecular generation.

Future work will focus on developing a more mechanism-aware molecular generation framework. Integrating target-related information, such as protein structures, drug-target interaction networks, protein-protein interaction data, and biological network representations, may help constrain generation toward molecules with more plausible binding mechanisms and disease relevance. Recent advances in biomedical knowledge graph learning, contrastive representation learning, molecular representation pre-training, molecular interaction prediction, knowledge-aware generative modeling, and transformer-powered biological network modeling provide useful directions for this extension [60–65]. In addition, progress in AI-assisted protein design, protein sequence representation learning, and computational drug development for protein targets could inform structure-aware prioritization and target-focused evaluation of generated molecules [66–68]. Future versions of AET5 may also combine retrosynthetic accessibility filtering, uncertainty estimation, and experimental feedback, forming a closed-loop workflow for expression-guided molecular generation and optimization.

## 5. Conclusion

This study presents AET5, an expression-conditioned molecular generation framework that integrates a conditional expression encoder with a fine-tuned MolT5 generator. By learning biologically informed conditional embeddings from gene expression profiles, AET5 can model disease-related cellular signals and guide molecular generation toward relevant chemical spaces. Compared with existing methods, AET5 achieves favorable performance in validity, novelty, diversity, and physicochemical property distributions. Case studies on SARS-CoV-2 and PC further provide computational evidence of its potential utility across both viral infection and cancer settings, suggesting its applicability for expression-guided molecular design.

Nevertheless, several limitations remain. The current validation is mainly computational, and the biological activity, safety, and target selectivity of generated molecules require experimental confirmation. In addition, target-level mechanistic information has not yet been explicitly incorporated into the generation process.

Future work will focus on integrating richer biological and target-related information to improve mechanistic control, interpretability, and candidate prioritization. Experimental validation and iterative feedback will also be important for further optimizing generated molecules. Overall, AET5 provides a robust and scalable framework for connecting transcriptomic profiles with the generation of biologically relevant molecular candidates.

## Supporting information

S1 Fig Comparison of ligand-protein binding sites between AET5-generated molecules and control molecules for the 2YCF target. (a) 3D interactions and actual binding sites of the generated molecule within the ligand-protein complex. (b) 2D interactions and actual binding sites of the generated molecule. (c) 3D interactions and actual binding sites of the control molecule within the ligand-protein complex. (d) 2D interactions and actual binding sites of the control molecule. (e) Comparison of binding energy distributions and numerical values. (f) Time-dependent comparison of the radius of gyration. (g) Time-dependent comparison of the number of hydrogen bonds. (h) Time-dependent comparison of RMSD.
(TIF)

S2 Fig Comparison of ligand-protein binding sites between AET5-generated molecules and control molecules for the 7LMD target. (a) 3D interactions and actual binding sites of the generated molecule within the ligand-protein complex. (b) 2D interactions and actual binding sites of the generated molecule. (c) 3D interactions and actual binding sites of the control molecule within the ligand-protein complex. (d) 2D interactions and actual binding sites of the control molecule. (e) Comparison of binding energy distributions and numerical values. (f) Time-dependent comparison of the radius of gyration. (g) Time-dependent comparison of the number of hydrogen bonds. (h) Time-dependent comparison of RMSD.
(TIF)

S3 Fig Comparison of ligand-protein binding sites between AET5-generated molecules and control molecules for the 9I1S target. (a) 3D interactions and actual binding sites of the generated molecule within the ligand-protein complex. (b) 2D interactions and actual binding sites of the generated molecule. (c) 3D interactions and actual binding sites of the control molecule within the ligand-protein complex. (d) 2D interactions and actual binding sites of the control molecule. (e) Comparison of binding energy distributions and numerical values. (f) Time-dependent comparison of the radius of gyration. (g) Time-dependent comparison of the number of hydrogen bonds. (h) Time-dependent comparison of RMSD.
(TIF)

S4 Fig Comparison of ligand-protein binding sites between AET5-generated molecules and control molecules for the 6AB2 target. (a) 3D interactions and actual binding sites of the generated molecule within the ligand-protein complex. (b) 2D interactions and actual binding sites of the generated molecule. (c) 3D interactions and actual binding sites of the control molecule within the ligand-protein complex. (d) 2D interactions and actual binding sites of the control molecule. (e) Comparison of binding energy distributions and numerical values. (f) Time-dependent comparison of the radius of gyration. (g) Time-dependent comparison of the number of hydrogen bonds. (h) Time-dependent comparison of RMSD.
(TIF)

S5 Fig Hyperparameter sensitivity analysis of AET5. (a, b) Effects of noise probability and noise strength on the final convergence behavior of encoder loss, generator training loss, and generator validation loss. (c, d) Effects of the temperature parameter in the encoder contrastive learning objective on contrastive loss and generator training loss. (e, f) Effects of generator decoding and sampling hyperparameters on molecular validity and internal diversity. (g, h) Effects of generator decoding and sampling hyperparameters on synthetic accessibility score and QED score.
(PNG)

S1 Table Ranking of gene attention during prostate cancer-guided molecular generation. Genes were selected from the union of the 978 landmark genes and known prostate cancer-associated genes. The rank indicates the model-assigned attention ranking among the 978 landmark genes. References support the reported association between each gene and prostate cancer.
(DOCX)
