# Supplementary material for: AET5: A transcriptome-guided molecular generation framework with contrastive self-supervised learning
Source: PLoS Comput Biol. 2026 Sep 10;22(9):e1014703. doi: 10.1371/journal.pcbi.1014703 (PMC13561366; doi:10.1371/journal.pcbi.1014703)
Supplement: Supplementary file 1 [file pcbi.1014703.s006.docx]

**Table S1:** Ranking of gene attention during prostate cancer-guided molecular generation.

| Gene Name | Confidence Score [1] | Rank/978 | Reference |
| --- | --- | --- | --- |
| *CHEK2* | 0.82 | 19 | [2] |
| *KAT6B* | 0.50 | 22 | [3] |
| *TOP2A* | 0.53 | 29 | [4] |
| *TUBB6* | 0.58 | 33 | [5] |
| *TP53* | 0.76 | 70 | [6] |
| *GATA2* | 0.61 | 71 | [7] |
| *RB1* | 0.61 | 92 | [8] |
| *CDKN1B* | 0.63 | 98 | [9] |
| *MSH6* | 0.59 | 109 | [10] |
| *MYC* | 0.57 | 117 | [11] |

**Reference**

1.	Ochoa D, Hercules A, Carmona M, Suveges D, Gonzalez-Uriarte A, Malangone C, et al. Open Targets Platform: supporting systematic drug-target identification and prioritisation. Nucleic Acids Research. 2021;49(D1):D1302-D10.

2.	Dong X, Wang L, Taniguchi K, Wang X, Cunningham JM, McDonnell SK, et al. Mutations in *CHEK2* Associated with Prostate Cancer Risk. The American Journal of Human Genetics. 2003;72(2):270-80.

3.	He W, Zhang M-G, Wang X-J, Zhong S, Shao Y, Zhu Y, et al. KAT5 and KAT6B are in positive regulation on cell proliferation of prostate cancer through PI3K-AKT signaling. International Journal of Clinical and Experimental Pathology. 2013;6(12):2864-71.

4.	Murphy AJ, Hughes CA, Barrett C, Magee H, Loftus B, O'Leary JJ, et al. Low-level TOP2A amplification in prostate cancer is associated with HER2 duplication, androgen resistance, and decreased survival. Cancer Research. 2007;67(6):2893-8.

5.	Jiang L, Zhu X, Yang H, Chen T, Lv K. Bioinformatics Analysis Discovers Microtubular Tubulin Beta 6 Class V (TUBB6) as a Potential Therapeutic Target in Glioblastoma. Frontiers in Genetics. 2020;11:566579.

6.	Schitcu VH, Raduly L, Zanoaga O, Jurj A, Munteanu VC, Budisan L, et al. TP53 gene implications in prostate cancer evolution: potential role in tumor classification. Medicine and Pharmacy Reports. 2023;96(4):384-91.

7.	Rodriguez-Bravo V, Carceles-Cordon M, Hoshida Y, Cordon-Cardo C, Galsky MD, Domingo-Domenech J. The role of GATA2 in lethal prostate cancer aggressiveness. Nature reviews Urology. 2017;14(1):38-48.

8.	Han W, Liu M, Han D, Li M, Toure AA, Wang Z, et al. RB1 loss in castration-resistant prostate cancer confers vulnerability to LSD1 inhibition. Oncogene. 2022;41(6):852-64.

9.	Chang B-l, Zheng SL, Isaacs SD, Wiley KE, Turner A, Li G, et al. A polymorphism in the CDKN1B gene is associated with increased risk of hereditary prostate cancer. Cancer Research. 2004;64(6):1997-9.

10.	Raymond VM, Mukherjee B, Wang F, Huang S-C, Stoffel EM, Kastrinos F, et al. Elevated Risk of Prostate Cancer Among Men With Lynch Syndrome. Journal of Clinical Oncology. 2013;31(14):1713-8.

11.	Koh CM, Bieberich CJ, Dang CV, Nelson WG, Yegnasubramanian S, De Marzo AM. MYC and Prostate Cancer. Genes & Cancer. 2010;1(6):617-28.
